# Explicit Scale Simulation for analysis of RNA-sequencing count data with ALDEx2

**DOI:** 10.1093/nargab/lqaf108

**Published:** 2025-08-19

**Authors:** Gregory B Gloor, Michelle Pistner Nixon, Justin D Silverman

**Affiliations:** Department of Biochemistry, University of Western Ontario, London ON, N6A 5C1, Canada; Department of Population Health Sciences, Geisinger, Danville, PA 17822, United States; College of Information Sciences and Technology, Pennsylvania State University, University Park, PA 16802, United States; Department of Statistics, Pennsylvania State University, University Park, PA 16802, United States; Department of Medicine, Pennsylvania State University, Hershey, PA 17033, United States

## Abstract

In high-throughput sequencing (HTS) studies, sample-to-sample variation in sequencing depth is driven by technical factors, and not by variation in the scale (size) of the biological system. Typically a statistical normalization removes unwanted technical variation in the data or the parameters of the model to enable differential abundance analyses. We recently showed that all normalizations make implicit assumptions about the unmeasured system scale and that errors in these assumptions can dramatically increase false positive and false negative rates. We demonstrated that these errors can be mitigated by accounting for uncertainty using a *scale model*, which we integrated into the ALDEx2 R package. This article provides new insights focusing on the application to transcriptomic analysis. We provide transcriptomic case studies demonstrating how scale models, rather than traditional normalizations, can reduce false positive and false negative rates in practice while enhancing the transparency and reproducibility of analyses. These scale models replace the need for dual cutoff approaches often used to address the disconnect between practical and statistical significance. We demonstrate the utility of scale models built based on known housekeeping genes in complex metatranscriptomic datasets. Thus this work provides guidance on how to incorporate scale into transcriptomic data sets.

## Introduction

High-throughput sequencing (HTS) is a ubiquitous tool used to explore many biological phenomenon such as gene expression (single-cell sequencing, RNA-sequencing, meta-transcriptomics), microbial community composition [16S ribosomal RNA (rRNA) gene sequencing, shotgun metagenomics], and differential enzyme activity (selex, CRISPR killing). HTS proceeds by taking a sample from the environment, making a library, multiplexing (merging) multiple libraries together, and then applying a sample of the multiplexed library to a flow cell. Each of these steps is a compositional sampling step as only a fixed-size subsample of nucleic acid is carried over to subsequent steps. Thus, with each sampling step the connection between the actual number of molecules in the sampled DNA pool and the environmental scale (e.g. total number of molecules, microbial load, or total gene expression) of the measured biological system is degraded or lost. In the end, the information contained in the data relates only to relative abundances and has an arbitrary scale imposed by the sequencing process [1–3].

The analysis of HTS data suffers from several known problems that can be traced, in whole or in part, to misspecification of scale in the output data. The first issue is poor control of the false discovery rate (FDR) [4, 4–8], exhibited as dataset-dependent FDR control which is observed as a disconnect between statistical and biological significance [8, 9]. In current practice, this issue is addressed by a dual-filtering method, whereby both a low *P*-value (or equivalently a low q-value following FDR correction [10]) and a large difference between groups is used to identify transcripts or genes of interest for follow-up analysis [9, 11]. This double-filtering approach is graphically exemplified by the volcano plot [11], but is known to not appropriately control the FDR [12, 13]. Since there is no standard way of determining what fold-change cutoff should be used researchers have unlimited degrees of freedom which is known to lead to unreliable inference [14]. In a recent example, Li *et al.* [8] used patient or clinically derived transcriptome datasets and found that many methods suffer from an extremely high false positive rate. The second issue is poor performance when analyzing data where the mean change between groups is nonzero [3]. Such asymmetric data can arise when a gene set is expressed in one group but not the other, or when one group contains different gene content from the other. This type of data frequently arises in *in-vitro* selection experiments (SELEX), transcriptome analysis, and microbiome analysis [15]. The third issue is that the actual scale of the environment is often a major confounding variable during analysis [3, 16]. This has long been known in transcriptome analysis and was a major driver for the development of normalization factors [17] and the use of molecular spike-ins to provide a reference set of known counts [18–21] to estimate scale. While often useful, spike-in methods only provide information downstream of the step in the sample preparation protocol where the intervention was made and introduce an additional source of variation that must be accounted for [19]. In the microbiome field, a recent landmark paper showed that biological scale was a major unacknowledged confounder in many human analyses [16]. These authors built a machine learning model to uncover the biological variation in scale and including this information was useful despite exhibiting only a modest correlation (estimated to be 0.6) with the scale of data external to the training set [16]. The final issue is that all the above problems become more pronounced as more samples are collected; that is, more information results in optimizing for a precise but inaccurate analysis [3, 8, 22].

The four problems were recently shown by Nixon *et al.* [3] to be a result of a mismatch between the underlying size or scale of the system and the assumptions of the normalizations used for the analysis of HTS. Biological variation in scale often represents an important unmeasured confounder in HTS analyses [15, 16, 19, 23]. For example, cells transformed by the cMyc oncogene have about 3 times the amount of mRNA and about twice the rRNA content than nontransformed cells [24], and this dramatically skews transcriptome analysis unless spike-in approaches are used [19]. In addition, wild-type and mutant strains of cell lines, yeast or bacteria have different growth rates and RNA contents under different conditions, which affect our ability to identify truly differentially abundant genes [25–27]. As another example, the total bacterial load of the vaginal microbiome differs by 1–2 orders of magnitude in absolute abundance between the healthy and bacterial vaginosis (BV) states [28], and the cell and RNA composition between these states is dramatically different [29, 30]. Thus, a full description of any of these systems includes both relative change (composition) and absolute abundance (scale). Current methods access only the compositional information yet make implicit assumptions about the scale [22].

Nixon *et al.* [3] showed that the challenge of nonbiological variation in sequencing depth could be explained as a problem of partially identified models. They showed that *all* normalizations make some assumption about scale but these implicit assumptions are often inappropriate and difficult to interpret. As a result, different normalizations provide different outputs when applied to the same dataset [4, 6, 17, 31, 32]. Intuitively, normalizations in widespread use assume that either all samples have the same scale, e.g. proportions, rarefaction [33], RPKM (reads per kilobase per million) [34, 35], etc; or that a subset of features in one sample can be chosen as a reference to which the others are scaled e.g. the TMM (trimmed mean of M values) [36], the LVHA (low variance, high abundance) [15], qPCR (quantitative PCR) [37], the additive log-ratio [38]; or that different sub-parts of each sample maintain a constant scale across samples e.g. the RLE (relative log-expression) [39]; or that the geometric mean of the parts is appropriate e.g the CLR (centred log-ratio) [40] and its derivatives. Notably when the assumption of similar scale across samples is not violated, or violated only weakly, any method will provide a reasonable analysis. The problem arises when this assumption is violated, in which case all tools fail without warning.

**Figure 1.**
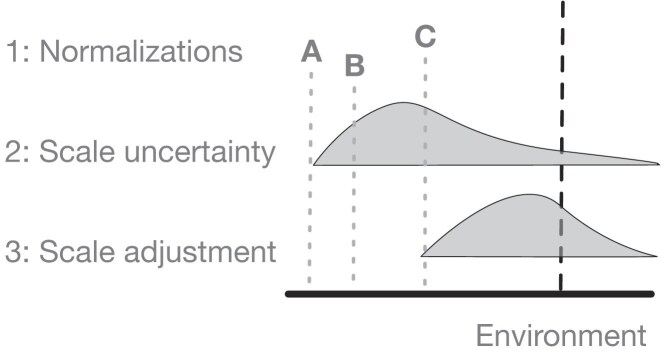
Mismatches between estimated scale and true scale lead to poor estimation of high throughput sequencing data. All normalizations used for differential abundance analysis make some strict assumption about the scale of the environment as shown in line 1. In this example all normalizations produce a biased estimate of the environmental scale, but estimate C is the closest to the truth. Adding uncertainty to normalization C as represented by the distribution in the line 2 leads to less bias and now includes the actual environmental scale in the assumption. As shown in line 3 in some cases it may be useful to adjust the center of the scale uncertainty estimate if the initial normalizations give very poor estimates of the underlying environment.

The original scale-naive ALDEx2 [41] model unwittingly made a strict assumption about scale through the CLR normalization and we found that the CLR was very sensitive to violations of the assumption of scale identity between groups [15]. Moreover, when the assumption of identity was not true the CLR used by ALDEx2 could be outperformed by other normalizations in simulation studies [42]. As illustrated graphically in Fig. 1, Nixon *et al.* [3] showed through simulation that introducing uncertainty into the scale assumption, and in more extreme cases altering the location of the scale assumption, resulted in more reproducible data analysis including better control of both false positive and false negative results. We modified ALDEx2 to explicitly model uncertainty in scale over a range of reasonable normalization parameters, and showed significant improvements in performance in microbiome and *in-vitro* selection experiments [22] and in a vaginal metatranscriptome analysis [43]. Here, we briefly review these modifications and show how adding scale uncertainty can greatly improve modeling in transcriptome and meta-transcriptome datasets to provide substantially more robust and reproducible results.

## Materials and methods

Formal and expanded descriptions of the concepts that follow are given in [3, 22]. To be concrete, we let **Y** denote the *measuredD* × *N* matrix of sequence counts with elements **Y**_*dn*_ indicating the number of measured DNA molecules mapping to feature *d* (e.g. a taxon, transcript or gene) in sample *n*. Likewise, we can denote **W**_*dn*_ as the *true* amount of class *d* in the biological system from which sample *n* was obtained. We can think of **W** as consisting of two parts, the scale **W**^*Tot*^ (e.g. totals) and the composition **W**^*Comp*^ (i.e. proportions). That is, **W**^*Tot*^ is a *N*-vector with elements $\mathbf {W}^{Tot}_{n}=\sum _{d}\mathbf {W}_{dn}$ while **W**^*Comp*^ is a *D* × *N* matrix with elements $\mathbf {W}^{Comp}_{dn}=\mathbf {W}_{dn}/\mathbf {W}^{Tot}_{n}$. Note that with these definitions **W** can be written as the element-wise combination of scale and composition: $\mathbf {W}_{dn}=\mathbf {W}^{Comp}_{dn}\mathbf {W}^{Tot}_{n}$, or as the logarithm $\log \mathbf {W}_{dn}= \log \mathbf {W}^{Comp}_{dn} + \log \mathbf {W}^{Tot}_{n}$.

Many of the normalizations used in tools such as DESeq2 [44], edgeR [36], metagenomeSeq [45], and ALDEx2 [46] can be stated as ratios of the form $\hat{{\mathbf {W}}}_{dn} \approx \mathbf {Y}_{dn}/f(\mathbf {Y})$, where the denominator is determined by some function of the observation. We use the hat notation ($\ \hat{}$) to indicate that the output is an estimate of the true value. The technical variation in sequencing depth, which is often called ‘library size’ has no relationship with the actual number of molecules in the sampled environment [1]. In other words ($\mathbf {Y}^{Tot}_{n}=\sum _{d}\mathbf {Y}_{dn}$) the observed data **Y** provides us with information about the system composition **W**^*Comp*^ but little to no information in the system scale **W**^*Tot*^ (Lovell *et al.*, 2011).

### Adding scale uncertainty in ALDEx2

The ALDEx2 R package [41, 46] is a general purpose toolbox to model the uncertainty of HTS data and to use that model to estimate the significance of the underlying log-fold change (LFC). At a high-level, ALDEx2 has three connected components to estimate the uncertainty inherent in HTS datasets. First, the tool accounts for the uncertainty of the sequencing counts using Dirichlet multinomial sampling to build a probabilistic model of the data; i.e. $\mathbf {\hat{W}}^{Comp} \approx \mathrm{Dir}(\mathbf {Y})$. Secondly, ALDEx2 uses the centered log-ratio transformation to scale the data [41]. It was this step that was modified to account for scale uncertainty and misspecification [22] explained with more details in [3, 22] and summarized in the next paragraph. Finally, a standard null-hypothesis test and a nonparametric estimate of mean standardized difference are used to report on the finite sample variation. These sources of uncertainty and variation are combined via reporting the expected values from a Monte Carlo simulation framework. For simplicity, we use the term ‘difference’ to refer to the absolute difference between groups, and ‘dispersion’ to refer to the within-condition difference or pooled variance as defined in [41]. These are calculated on a log_2_ scale. For more details on ALDEx2 see [3, 22, 41, 46].

Scale models were incorporated into ALDEx2, turning the ALDEx2 model into a specialized type of statistical model which Nixon *et al.* [3] term a *Scale Simulation Random Variable* (SSRV). To do this, Nixon *et al.* [3] generalized the concept of normalizations by introducing the concept of a *scale model* to account for potential error in the centered log-ratio normalization step. They did this by including a model for $\mathbf {\hat{W}}^{Tot}_{n}$. The CLR normalization used by ALDEx2 makes the assumption $\mathbf {\hat{W}}^{Tot}_{n}=1/G_{n}$, where *G*_*n*_ is the geometric mean of the counts (or the corresponding proportions)of each part in sample n, which while being a random variable, is essentially constant across each Monte Carlo replicate, but that differs between samples. With this modification, ALDEx2 can be generalized by considering probability models for the scale $\mathbf {\hat{W}}^{Tot}_{n}$ that have mean 1/*G*_*n*_. For example, the following scale model generalizes the CLR:


\begin{eqnarray*}
\log \mathbf {\hat{W}}^{Tot}_{n} = -\log G_{n} + \Lambda x_{n} \qquad \Lambda \sim N(\mu , \gamma ^{2}).
\end{eqnarray*}

This formulation is quite flexible [3, 22]. In the simple or ‘default’ configuration, μ = 0 and γ is a tunable parameter drawn from a log-Normal distribution [3]. Adding scale uncertainty with the γ paramenter [as shown in Fig. 1(1)] controls only the degree of uncertainty of the CLR assumption for the *x*_*n*_ binary condition indicator (e.g. *x*_*n*_ = 1 denotes case and *x*_*n*_ = 0 denotes control). However, the default model does not change the location of the default scale estimate. In the advanced or ‘informed’ configuration, μ takes different values for each group and controls the location of the LFC assumption [as shown in Fig. 1(2)]; combining μ with a γ estimate allows for uncertainty in both the location and the scale. In the manuscript where the idea of scale was original derived, Nixon *et al.* [3] conducted extensive simulations showing that the default model had increased specificity and that the informed approach exhibited increased sensitivity and specificity [22]. In this report we show that these approaches also work well to control the FDR in transcriptome and metatranscriptome datasets. We also provide some additional insights into how scale models achieve this outcome. These modifications are instantiated in ALDEx2 which is the first software package designed for SSRV-based inference.

## Results

### Adding scale uncertainty replaces the need for dual significance cutoffs

Gierliński *et al.* [47] generated a highly replicated yeast transcriptome dataset to compare gene expression between a wild-type strain and a snf2 gene knockout, Δsnf2. This dataset of 86 samples (44 and 42 per group) is an example of technical growth replicate experiments common in the literature. The dataset was used to test several RNA-seq tools for their power to detect the set of differentially abundant transcripts identified in the full dataset when the data was subset [9]. In this original study each tool had its own ‘gold standard’ set of transcripts with different tools identifying between 65% and >80% of all transcripts as being significantly different. Since the majority of transcripts were significantly different, the authors suggested that it was more appropriate to apply a dual cutoff composed of both a Benjamini–Hochberg [48] corrected *P*-value (q-value) plus a fold-change, or difference, cutoff to limit the number of identified transcripts to a much smaller fraction of the total. In other datasets, Nixon *et al.* [22] showed that adding even a small amount of scale uncertainty with ALDEx2 dramatically reduced the number of significant transcripts identified, removing the need for the dual cutoff approach in this dataset and others.

We start with the assumption that not all statistically significant differences are biologically relevant [49], and that a result where the majority of transcripts are significant breaks the necessary assumption for DA/DE (differential abundance or expression) expression that most parts be invariant [31]. Transcriptomic analysis commonly uses a dual cutoff approach graphically exemplified by volcano plots [9, 11]. Using either DESeq2 or ALDEx2, a majority of transcripts are statistically significantly different between groups with a q-value cutoff of ≤0.05; i.e. 4636 (79%, DESeq2) or 4172 (71%, ALDEx2) of the 5891 transcripts. These values are in line with those observed by [9]. Such large numbers of statistically significant transcripts seems biologically unrealistic. That 118 transcripts are identified by ALDEx2 and not DESeq2, while DESeq2 identifies 582 transcripts that ALDEx2 does not, suggests that the choice of normalization plays a role in which results are returned as significant and that some, if not the majority, are driven by technical differences in the analysis [8, 31] or are false positives.

**Figure 2.**
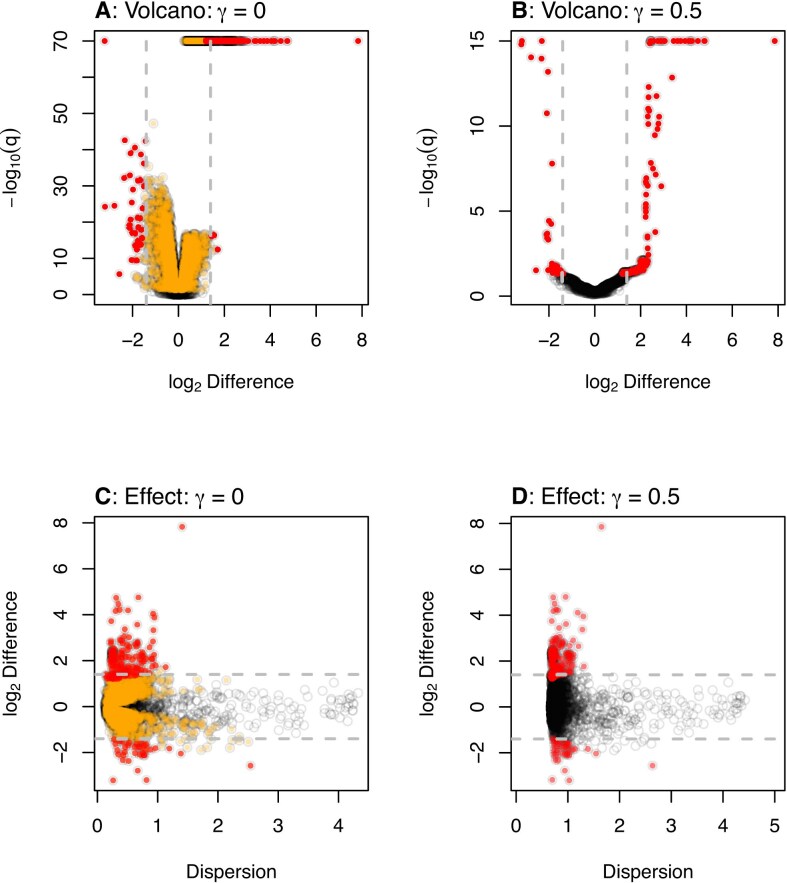
Volcano and effect plots for unscaled and scaled transcriptome analysis. ALDEx2 was used to conduct a differential expression analysis on the yeast transcriptome dataset. The results were plotted to show the relationship between difference and dispersion using effect plots or difference and the q-values using volcano plots. (**A**, **C**) For the naive analyses. (**B**, **D**) For the default analyses that include scale uncertainty. Each point represents the values for one transcript, with the color indicating if that transcript was significant in the both analyses (red) or in the naive analysis only (orange). Points in gray are not statistically signficantly different under any condition. The horizontal dashed lines represent a log_2_ difference of ±1.4.

The volcano plots in Fig. 2A and B show that adding scale uncertainty increases the minimum q-value and increases the concordance between the q-value and the difference between groups [compare panels (A) and (B)]. The effect plots [50] in Fig. 2C shows that the majority of significant transcripts (red, orange) have negligible differences between groups and very low dispersion. We suggest that this low dispersion is driven by the experimental design which is actually a technical wet lab replication rather than a true biological replication design [47]. Scale uncertainty can be incorporated using the gamma parameter that controls the amount of noise added to the CLR mean assumption when we call either aldex(), or aldex.clr(). Figure 2B and D shows that setting γ = 0.5, i.e. adding 0.5 standard deviation (SD) of uncertainty, now results in 205 which is far fewer statistically significant transcripts than in the naive analysis and we observe that the minimum dispersion increases from 0.12 (γ = 0 ) to 0.67 (γ = 0.5).

It is common practice to use a dual-cutoff by choosing transcripts based on a thresholds for both q-values and fold-changes [9], and these were first proposed for microarray experiments through volcano plots [11]. Note that there is considerable variation in recommended cutoff values [9], and that this controversy has persisted ever since fold-change was suggested [51]. Unfortunately, universal cutoff fold-change values cannot be identified in part because different tools have intrinsically different variance in their log_2_-fold change ranges [52]. This has led to the widespread practice of applying a *post-hoc* fold-change cutoff to reduce the number of positive identifications to a manageable proportion of the whole dataset. Here, we applied the dual-cutoff method using a fold-change of at least a 2^1.4^ fold change that reduces the number of significant outputs to 193 for DESeq2 and to 186 for ALDEx2. This cutoff was chosen for convenience and is mid-way between the high and low fold-change recommendations of [9]. These limits are shown by the dashed gray lines in Fig. 2 and we can see that a 2^1.4^ fold change ( ∼ 2.6 fold) cutoff identifies a similar number of transcripts as does ALDEx2 using γ = 0.5 which identifies 205 transcripts.

Supplementary Fig. S1 shows an example of the aldex.senAnalysis() function to identify those transcripts that are very sensitive to scale uncertainty in this dataset. Here we see that adding a very small amount of scale γ = 0.1 reduces the number of significant transcripts by more than half in the yeast dataset. This allows the analyst to ignore those low-dispersion transcripts that were significant only because of an absence of scale uncertainty.

We next examined how adding scale would alter the analysis in a real dataset to which synthetically generated true positive (TP) counts had been added. We show results from the anti-PD-1 therapy RNA-seq dataset [53] which examined changes in gene expression when cells were exposed or not to a cell-cycle checkpoint inhibitor. This dataset was used by Li *et al.* [8] as an example of the dangers of relying on tools with high false positive error rates when analyzing clinical or clinically related transcriptome samples. Indeed, in the benchmarking analysis done by this group, they found that parameter-based methods such as DESeq2 and edgeR that rely on reported *P*-value and fold-change cutoffs often led to conclusions in the original dataset that were indistinguishable from permutations of the dataset. In other words, that the analysis of transcriptome datasets from patient-derived samples often exhibited many false-positive identifications.

**Figure 3.**
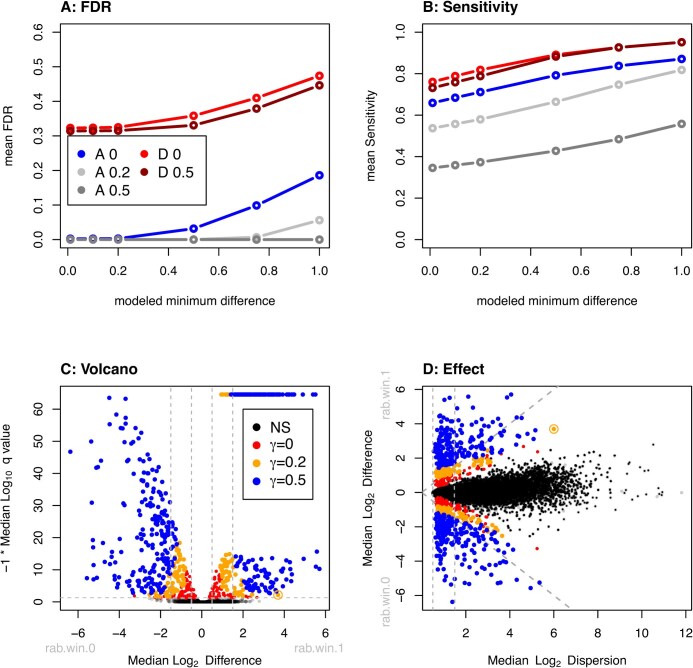
Results of modeling TP differences in the PD-1 dataset. For this the data were shuffled and 5% of the transcripts were modeled to have TP differences between groups where the differences were drawn from a Normal distribution with a mean difference of 0 and a SD of 2. (**A**) Shows the mean FDR of 10 instances for scale-naive ALDEx2 (A 0), and ALDEx2 with γ = 0.2 (A 0.2) or γ = 0.2 (A 0.5), and where significant features were identified by DESeq2 without (D 0), or with (D 0.5) a 0.5-fold change difference. The *x*-axis shows how the FDR changes for each tool as a function of the estimated modeled difference between groups. (**B**) Shows the mean sensitivity (TP found/all TP). (**C**, **D**) Show volcano and effect plots for the ALDEx2 output with the transcripts identified as significant at each scale setting as colored points, and nonsignificant transcripts in black.

Figure 3 shows the results of ten permutations of this dataset while simulating 5% of the transcripts to be TPs where the difference from no change was derived from a Normal distribution [54]. This simulation was conducted with the seqgendiff R package [54] to both permute the dataset and to add TP features. We did ten permutations and kept track of the number of true and false positive identifications. Figure 3A shows the actual FDR for ALDEx2 with and without the addition of scale uncertainty and for DESeq2 with or without a fold-change cutoff of 2^0.5^. When the modeled difference was >0, ALDEx2 exhibited a FDR of γ = 0: 0.002, γ = 0.2: 0, and γ = 0.5: 0, while DESeq2 with no fold-change cutoff had an FDR of 0.32 and with a 0.5-fold-change cutoff an FDR of 0.31. Figure 3 also plots these results as a function of the minimum modeled fold change of the TP transcripts. First, we can see that this analysis recapitulates the observations of Li *et al.* [8] in that DESeq2 has very poor false positive control at a nominal FDR of 0.05. The FDR is not controlled any better when a fold-change cutoff is applied, and this agrees with previous work showing that fold-change cutoffs do not materially improve FDR control in high throughput datasets [12, 13]. Figure 3B shows that DESeq2 has higher sensitivity than does ALDEx2, and not surprisingly this sensitivity increases as the modeled minimum difference between groups increases. For the case where the mean difference was 0 or greater, ALDEx2 exhibited a sensitivity of γ = 0: 0.66, γ = 0.2: 0.54, and γ = 0.5: 0.35, while DESeq2 with no fold-change cutoff had a sensitivity of 0.76 and with a 0.5-fold-change cutoff a sensitivity of 0.73. In this example, scale-naive ALDEx2 has near perfect FDR control and reasonable sensitivity at low modeled difference between groups. However, when the modeled difference between groups becomes large, then the scale-naive version of ALDEx2 begins to exhibit unacceptable rates of false positives and the false positive rate for DESeq2 also increases to nearly 50%. Thus, even though sensitivity is nearly absolute, at a nominal FDR of 0.05, whether a transcript is actually differentially expressed is essentially a coin flip. In contrast, adding in even small amounts of scale uncertainty (0.2 SD) with ALDEx2 drops the true FDR rate to 0, but at the expense of some sensitivity. The major contributor to the increase in FDR with larger modeled differences is that the tools are identifying as positives those transcripts that are modeled to have differences just below the threshold. We need to recognize that there is no such thing as a statistical free lunch; the analyst can have high sensitivity but low confidence that any individual transcript is truly different, or have lower sensitivity but have very high confidence that the difference is real. In other words, the sensitivity of a method is directly tied to how much error the investigator is willing to tolerate.

Examination of the volcano plot [11] and effect plot [50] in Fig. 3C and D provides some insight into why adding scale uncertainty provides better FDR control than does the approach of using a *P*-value and a fold-change cutoff. In the volcano plot, adding scale uncertainty differentially excludes transcripts with a combination of marginal *P*-values and low difference between groups, and that this becomes more pronounced with a larger scale value. This can be most clearly seen in panel (C), where the red (excluded by scale uncertainty of 0.2), and orange (excluded by scale uncertainty of 0.5) exhibit boundaries that are not vertical but are angled. This effect is also seen in Fig. 2 but is more nuanced because of the very low intrinsic dispersion in this dataset. In contrast, the fold-change cutoff only includes the magnitude of change, and not the size of the *P*-value and so transcripts with large differences, but marginal *P*-values are retained. The effect plot in Fig. 3D shows that the transcripts with marginal *P*-values and large differences that are excluded when scale uncertainty is added are those that have a large dispersion.

As a concrete example consider the point that is circled in panels (C) and (D) with a marginal *P*-value, with a difference between of nearly 4 and a dispersion of >6. This transcript is no longer significant when γ = 0.2, but would require a very large fold-change cutoff to be excluded by the standard approach. In addition, transcripts with very small dispersion and very small differences are also excluded when scale uncertainty is added. Thus, the addition of scale uncertainty achieves the desired outcome of lowering the FDR for those transcripts that are either marginally differentially abundant, or where the underlying dispersion—and hence the uncertainty in measurement—is very high, or for transcripts that fit both criteria.

Supplementary Fig. S2 shows a second permuted dataset with the addition of modeled differences between groups. Here we used another real dataset with over 200 biological replicates of BRCA1 tumor and control tissue samples from Li *et al.* [8]. This supplementary figure shows that the FDR control of DESeq2 is somewhat better than in the PD-1 dataset, although still much greater than the the nominal 5%. Further, a fold-change cutoff reduces the FDR of DESeq2 from about 30% to just over 20% with a power of over 80%. However, we can see that scale-naive ALDEx2 performs substantially better with a negligible FDR and comparable power. Adding scale uncertainty again improves FDR even for those transcripts modeled to have larger differences and the power is substantially better than in the PD-1 dataset, reaching the same power as DESeq2 or scale-naive ALDEx2 when the modeled difference is large. As before, this is driven by removing from consideration those transcripts with either a small difference between or a marginal *P*-value, or both.

Supplementary Fig. S3 shows that effect of applying γ = 0.5 to this dataset results in reducing the number of positive transcripts from being ∼70% of the whole dataset to <10% of the dataset and that this is largely because of a reduction in significance of those transcripts with low dispersion. Supplementary Fig. S4 shows a sensitivity analysis of the BRCA1 dataset showing that different scale uncertainty amounts alter the number of significant transcripts in a biological replicate experiment similarly to a technical replicate experiment. Supplementary Fig.s S5 and S6 delve into how adding scale uncertainty affects the variance–abundance relationship in subtle ways, and may help readers to understand the observations seen in Fig. 3 and Supplementary Fig. S2.

Together the results in this section show that adding scale uncertainty has the desirable effect of altering the transcripts identified as significantly different between groups in a way that exhibits better control of FDR albeit with a corresponding reduction in sensitivity. Those parts that were statistically significantly different *only because of low dispersion* or that *had marginal P-values* or both, are now preferentially excluded from statistical significance. In practice, we suggest that a gamma parameter of between 0.2 and 0.5 is realistic for most experimental designs [22] regardless if the replication is technical or biological.

### Housekeeping genes and functions to guide scale model choices

Dos Santos *et al.* [55] used a vaginal metatranscriptome dataset to compare the gene expression in bacteria collected from healthy (H) and BV affected women. This dataset is derived from two publicly available datasets composed of a set of 20 nonpregnant women from London, Ontario Canada [56], and a subset of 22 nonpregnant women collected from German women who underwent metronidazole treatment for BV [57]. Batch effects for these two groups were removed with ComBat-seq [58] and the two datasets were merged into one, giving a total of 16 H and 26 BV samples. In the Dos Santos paper, all results from this initial analysis were replicated in a much larger dataset derived from the MOMS-PI study [59].

In this vaginal environment, both the relative abundance of species between groups and the gene expression level within a species is different [60]. Additionally, prior research suggests that the total number of bacteria is about 10 times more in the BV than in the H condition [28]. Thus, these are extremely challenging datasets in which to determine differential abundance as there are both compositional and scale changes between conditions. The usual method to analyze vaginal metratranscriptome data is to do so on an organism-by-organism basis [57, 59, 60] because the scale confounding of the environment is less pronounced. One attempt at system-wide analysis returned several housekeeping functions as differentially expressed between groups [57]; a result likely due to a disconnect between the assumptions of the normalization used and the actual scale of the environment [15].

In this example, we show how to specify and interpret a user defined or *informed* scale model that can explicitly account for some of these modeling difficulties [22] even in a difficult to analyze dataset. An informed scale model can control for both the mean difference of scale between groups (e.g. directly incorporate information on the differences in total number of bacteria between the BV and H conditions) as well as the uncertainty of that difference as illustrated in Fig. 1(3). To specify a user-defined scale model, we can pass a matrix of scale values instead of an estimate of just the scale uncertainty to aldex.clr(). This matrix should have the same number of rows as the of Monte Carlo Dirichlet samples, and the same number of columns as the number of samples. While this matrix can be computed from scratch by the analyst, there is an aldex.makeScaleModel() function that can be used to simplify this step in most cases. This encodes the scale model as Λ ∼ *N*(*log*_2_μ_*n*_, γ^2^), where μ_*n*_ represents the scale value for each sample or group and *gamma* is the uncertainty as before. The scale estimate can be a measured value (cell count, nucleic acid input, spike-ins, etc) or an estimate. Nixon *et al.* [3, 22] showed that only the ratio of the means are important when providing values for μ_*n*_; i.e. the ratio between the log_2_μ_*i*_ and log_2_μ_*j*_ values. See the supplement to Nixon *et al.* [22] for more information.

**Figure 4.**
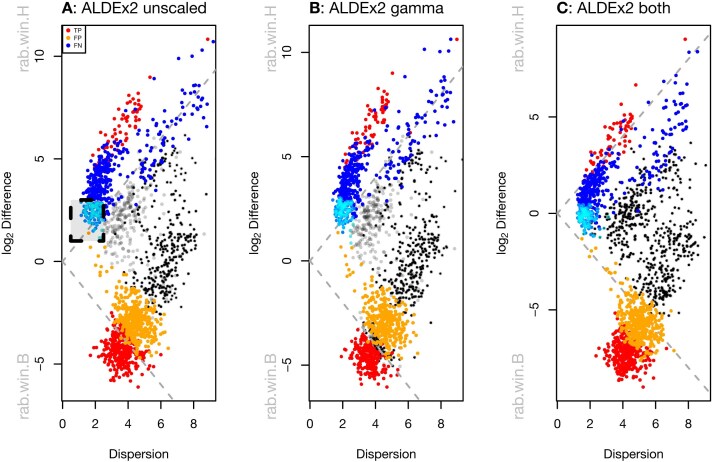
Analysis of vaginal transcriptome data aggregated at the Kegg Orthology (KO) functional level. (**A**) Shows an effect plot for the default analysis where the functions that are elevated in the healthy individuals have positive values and functions that are elevated in BV have negative values. Highlighted in the box are KOs that are almost exclusively housekeeping functions; these and functions with similar dispersion/difference relationships are colored cyan. These housekeeping functions should be located on the midline of no difference. (**B**) Shows the same data scaled with γ = 0.5, which increase the minimum dispersion as before. (**C**) Shows the same data scaled with γ = 0.5 and a 0.14-fold difference in dispersion applied to the BV samples relative to the H samples. In these plots statistically significant (q-value < 0.01) functions in the informed model are in red, false positive functions are in blue, nonsignificant functions in black, and false negative functions are in orange.

Figure 4A shows an effect plot of the data where reads are grouped by homologous function regardless of the organism of origin. Each point represents one of 3728 KEGG functions [61]. There are many more functions represented in the BV group (bottom) than in the healthy group (top). This is because the *Lactobacilli* that dominate a healthy vaginal microbiome have reduced genome content relative to the anaerobic organisms that dominate in BV, because there is a greater diversity of organisms in BV than in H samples, and because the BV condition has about an order of magnitude more bacteria than does the H condition.

The naive scale model appears to be reflecting the bacterial load as observed by calculating the mean scale value for each group. When using scale models with the aldex.clr function, the scale model is saved as a matrix in the @scaleSamps slot of the resulting object. Taking the mean of the rows of this matrix returns the scale estimates for each sample. We can thus determine the naive scale model by setting γ = 1*e* − 3. The naive scale estimate for the healthy group is 17.41 and for the BV group is 14.59 for a difference of 2.82 using base 2 for the logarithm. This is interpreted as the scale of the H group of samples being 7.06-fold greater than the BV group.

Applying the default scale model by including only uncertainty in the scale by setting γ = 0.5 increases the dispersion slightly but does not move the housekeeping functions toward the midline. This is as expected; the mean of the default scale model is based on the CLR normalization so no shift in location is expected over the scale-naive ALDEx2 model. Nevertheless, about 30% of the housekeeping functions are no longer statistically significantly different. Note that this change is simple to conduct, has no additional computational complexity and requires only a slight modification for the analyst.

There are 101 functions with low dispersion that appear to be shared by both groups (boxed area in Fig. 3A, and colored in cyan). Inspection shows that these largely correspond to core metabolic functions such as transcription, translation, ribosomal functions, glycolysis, replication, chaperones, etc (Supplementary file housekeeping.txt). The transcripts of many of these are commonly used as invariant reference sequences in wet lab experiments [62] and so are not be expected to contribute to differences in ecosystem behavior. Because we expect housekeeping functions to be nearly invariant in their expression and to occur in all organisms, the average location of these should be centered on 0 difference to represent an internal reference set. However, with the naive scale model, the mean difference of these housekeeping functions is approximately located at 2.3. Thus, we desire a scale model that approximately centers the housekeeping functions; thus an appropriate informed scale model in this dataset for functional analysis will place these functions closer to 0 than does the naive estimate. One way to choose an appropriate value for μ_*n*_ is to use the aldex.clr function on only the presumed invariant functions setting γ > 0, and then accessing the @scaleSamps slot as before. Doing so suggests that the difference in scale should be about 14%. A second approach would be to identify the functions used as the denominator with the denom=‘lvha’ option [15] for the aldex.clr function, and then to use these values as before. This approach suggests a 5% difference in scale, and is potentially less subject to user interpretation.

For the purposes of this example, if we assume a 14% difference in scale, we can set μ_*i*_ = 1 and μ_*j*_ = 1.14 using the makeScaleMatrix function. This function uses a logNormal distribution to build a scale matrix given a user-specified mean difference between groups and uncertainty level. Applying a per-group relative differential scale of 0.14 moves the housekeeping functions close to the midline of no difference (Fig. 3C, assuming 14% mean difference = −0.24, assuming a 5% mean difference = −0.34), and adding some uncertainty using a gamma of 0.5 provides the same dispersion as in panel (B) of Fig. 3. Note that now a significant number of functions are differentially up in BV that were formerly classed as not different without the full scale model (orange), or when only a default scale was applied. Inspection of the functions shows that these are largely missing from the *Lactobacillus* species and so should actually be captured as differentially abundant in the BV group. Supplementary Fig. S7 shows that the using either the 5% or the 14% scale difference give imperceptibly different results suggesting that an informed scale model that includes some uncertainty does not have to precisely estimate the scale difference to be useful. Nixon *et al*, [22] also found that multiple reasonable estimates for the μ_*n*_ part of the informed scale model were similarly useful in microbiome data.

Thus, applying an informed scale allows us to distinguish between both false positives (housekeeping functions in cyan, and others in blue) and false negatives (orange functions) even in a very difficult to analyze dataset. We used this informed scale model to uncover hither-to-now unknown differences in microbiome functional activity between the Healthy and BV cohorts that were missed in previous analyses and that explain important clinical differences between them [55]. The remarkable improvements in biological interpretation afforded by an informed scale model, and the transferrability of it between sample cohorts of the same condition is outlined in dos Santos *et al.* [55]. We suggest that the default scale model is sufficient when the data are approximately centered but that an informed model is more appropriate with datasets are not well centered or when the investigator has prior information about the underlying biology.

## Discussion

Scale estimates affect two parts of the analysis. Modeling uncertainty in scale prevents false certainty in the precision of estimation and controls false positive identification [3]. Modeling between-group scale differences relaxes the assumption of identity between the sizes of the environments made by many normalizations and allows better control of false negative identification. The scale estimates can be derived from the total number of molecules in the environment or from other estimates of input size (cell counts, initial concentrations, spike-ins, growth rates, etc).

Biological systems are both predictably variable and stochastic [63] and systems biology experiments show that there are transcripts with approximately constant concentrations in the cell and those with large variability under different growth conditions [25]. Current measurement methods that rely on high throughput sequencing fail to capture all of the variation, particularly variation due to scale and our uncertainty in measuring it [3, 22]. In the absence of external information [19, 20, 64] sequencing depth normalization methods cannot recapture the scale information [19, 23], and can only normalize for the technical variation due to sequencing depth. Here we demonstrated that even approximate estimates of the true system scale and the uncertainty of measuring it can aid in the interpretation of RNA-sequencing experiments.

Nixon *et al.* [3] introduced the idea of explicitly modeling the scale of a HTS dataset, and showed how to incorporate these models in the analysis of microbiome and other datasets [22]. They demonstrated that many tools commonly used to analyze HTS datasets had substantial Type 1 and Type 2 error rates in line with recent findings by others [5, 7, 8]. A version of ALDEx2 with the ability to include scale uncertainty was shown to be able to correct for high Type 1 error rate for that tool, albeit with some loss of sensitivity. Finally, they showed that incorporating an informed scale model incorporating both location and scale uncertainty estimates could both control for Type 1 and Type 2 error rates [22, 43].

The process of choosing the parameters depends on the analysis. If the scale of the groups is relatively constant then using a default scale model choosing γ = 0.5 will result in excellent FDR control, but at the loss of sensitivity when the difference between groups is <1. If the analyst is interested in smaller differences then setting γ = 0.2 may be more appropriate. The parameters for informed scale models are experiment-specific and can be anchored in known information such as cell counts, spike-ins, information from the literature or similar [3, 22, 65], and we recommend also including scale uncertainty as outlined for the default model. In the yeast and in the shuffled clinical transcriptome datasets, we made the assumption that the underlying biological scale was approximately constant and so used only the default scale model that includes only uncertainty in the scale of the sampled environments. Here, we observed the even moderate amounts of scale uncertainty led to much better control of the FDR than did simple fold-change cutoffs. We also observed a loss of sensitivity with the default scale model, but showed that this was because of differential exclusion of those transcripts with marginal *P*-values. In the metatranscriptome example [43] the choice of parameter for the difference in scale between groups was driven by the assumption inherent in the biology that core housekeeping functions would serve as an appropriate standard [37]. Thus, the choice of the μ parameter should be guided by the experimental question and the assumptions of the investigator. The used of informed scale models ensures that all assumptions are available for the reader.

Building and using a scale model thus has substantial benefits relative to the dual cutoff approach that is advocated for many gene expression experiments [9, 11]. In particular, the dual cutoff approach has long been known to not control for Type 1 errors [12, 13], and the frequent lack of concordance between tools when benchmarked on transcriptomes [5, 7–9, 17, 66] and microbiomes [4, 6, 32, 42, 67, 68] suggests poor control of Type 2 errors as well [5, 8]. Thus, incorporating a scale model during the analysis of HTS data promises the best of both worlds. A default scale model can control for Type 1 errors with minimal prior knowledge of the environment and this can be done with essentially no additional computational overhead. It must be acknowledged that this Type 2 error control comes at some expense of sensitivity, especially when the difference between groups is very small. Furthermore, this work and previous [22, 43] show that even minimal information about the underlying environment can be used to build a relatively robust informed scale model that controls for both Types 1 and 2 error rates. However, modeling and theory suggests that the inclusion of any reasonable amount of scale uncertainty is guaranteed to result in substantially better controlling the FDR [3, 22].

It is important to note that the approach advocated here is distinct from that suggested by Zhang *et al.* [69, 70] where the DNA amount for a gene is a covariate in the model for transcriptomic differential abundance. In our analysis we grouped all the transcript information to functional level regardless of organism, instead of modeling per-organism gene abundances. In the future we anticipate being able to build more complex models similar to those used by Zhang *et al.* with the additional information of uncertainty in the underlying gene count.

In the analysis of HTS data it is often observed that larger datasets converge on the majority of parts being significantly different [3, 8, 9]. Li *et al.* [8] conducted a permutation-based benchmarking study and found that widely used tools performed worse than simple Wilcoxon rank-sum tests coupled with the TPM (transcripts per million) normalization in controlling the FDR when sample sizes became large. Li *et al.* suggested that the presence of outliers were one of the factors driving the extreme FDR in some tests. We found that when the Wilcoxon test was used within the ALDEx2 framework that it had essentially the same outputs as did the *t*-test. For example, in the PD-1 dataset where γ = 0 the ALDEx2 *t*-test exhibited a mean FDR of 0.2% and mean sensitivity of 65.9% while the corresponding values from the ALDEx2 Wilcoxon test were 0.3% and 68.9%. This result again supports that the assumptions of the normalizations are as important or more important than the statistical test. Brooks *et al.* [71] suggested that inappropriate choice of benchmarking methods are also a major contributing factor and that better objective standards of truth are needed. In this report we generated semi-synthetic test data used binomial thinning which produces data that more closely mimic the properties of real high throughput sequencing data, and so can more rigorously test different tools [54]. From the perspective of our work the disagreement between tools can be explained by the observation that different analytic approaches produce different parameter estimates for either location or scale, or for both, as suggested in Fig. 1. Thus, more data produces worse estimates because the additional data simply increases the precision of a flawed estimate while incorporating uncertainty in the scale at least guarantees that we are less wrong [3, 72].

Scale simulation is now built into ALDEx2 [22] and in this report we suggest that there are two main root causes to common HTS data pathologies. The first contributing factor is the observed very low dispersion estimate for many features that is a by-product of some experimental designs and normalizations (Fig. 2). Supplying additional uncertainty alleviates many FP (false positives) but in a way that more appropriately controls the FDR as shown in Fig. 3. The second contributing factor is unacknowledged asymmetry in many datasets [15]; i.e. different gene content or a directional change in the majority of features. In the case of asymmetry, the use of a user-specified scale model can be very useful for otherwise difficult-to-analyze datasets such as meta-transcriptomes and *in-vitro* selection datasets where the majority of features can change as shown in Fig. 4. We showed two ways of estimating the scale difference between groups and found that any reasonable estimate is an improvement over the naive approach and also over the default scale model. This is in line with the observations by Nixon *et al.* [22] in a 16S rRNA gene sequencing dataset. While we acknowledge that some prior information is needed that this information is widely available and is already used when performing the gold-standard quantitative polymerase chain reaction test of differential abundance [73, 74].

Beyond concerns of fidelity and rigor, scale models also enhance the reproducibility and transparency of HTS analyses. The development of HTS and the associated problems of very high dimensional data that was not always statistically well-behaved led to many different proposed solutions including multiple normalizations and moderated test statistics. That these perform poorly in real data is shown by the simulation data in this report and elsewhere where both DESeq2 (which we used) and edgeR (which uses moderated statistical tests) performed poorly in controlling the FDR [8]. While these approaches often work in many datasets they fail to address the underlying problems of information that is missing in the data which is what is supplied by adding uncertainty around the information we have about that data. The addition of scale uncertainty directly addresses the missing information by testing the model over a range of normalizations [3]. In doing so, the scale-based approach removes the need for moderated statistics and can replace the consensus approach that has been proposed by some groups [6, 75] with no additional computational overhead. Thus, an advantage of incorporating scale is that analyses can be made much more robust such that actual or potential differences in scale can be tested and accounted for explicitly. While it is beyond the scope of the present article, we note that there are many ways of building scale models that enhance the interpretability of the parameters and assumptions and a detailed description of these points is describe elsewhere [3].

In summary, we supply a toolkit that makes incorporating scale uncertainty and location information simple to incorporate for transcriptomes or indeed any type of HTS dataset. While the underlying scale of the system is generally inaccessible, the effect of scale uncertainty on the analysis outcomes can be modeled and can help explain some of the underlying biology. Adding scale information to the analysis allows for more robust inference because the features that are sensitive to scale can be identified and their impact on conclusions weighted accordingly. The use of informed scale models permits difficult to analyze datasets to be examined in a robust and principled manner even when the majority of features are asymmetrically distributed or expressed (or both) in the groups [55]. Thus, using and reporting scale uncertainty should become a standard practice in the analysis of HTS datasets.

## Supplementary Material

lqaf108_Supplemental_Files

## Data Availability

The code is available at https://github.com/ggloor/scale-sim-bio. Materials, code, and figures are available at https://doi.org/10.6084/m9.figshare.29508986.
