# Supplementary material for: Explicit Scale Simulation for analysis of RNA-sequencing count data with ALDEx2
Source: NAR Genom Bioinform. 2025 Aug 19;7(3):lqaf108. doi: 10.1093/nargab/lqaf108 (PMC12362245; doi:10.1093/nargab/lqaf108)
Supplement: lqaf108_Supplemental_Files [file lqaf108_supplemental_files.zip › RPA_PolA SI_4.pdf]

Supplementary Materials

for the article

Human primosome requires replication protein A when copying DNA with inverted repeats

Andrey G. Baranovskiy[1], Lucia M. Morstadt[1], Eduardo E. Romero[2,3,4], Nigar D. Babayeva[1], Tahir H. Tahirov[1*]

Correspondence to: ttahirov@unmc.edu

This file includes:

Supplemental Tables S1 and S2

Supplemental Figures S1 – S10

Legend for Supplemental Movie S1

Supplemental Table S1. Oligonucleotides used in this study

Name	Sequence [a]	Application	Length
T1	ATTTTGAAGAGAAATACTTTAAATCAGTCTGGAATGATGAAGATTACTAGTGA AGATTCTGAGCGTCTTAATCTAAGTACAGGTCGTGCCGCCAAAAA[b]	Primer extension	98
T2	ATTTTGAAGAGAAATACTTTAAATCAAATGATGAAGATATCTAATCTATGGTCG CTCCATTCAGGAGCGACCTGTAGTACAGGTCGTGCCGCCAAAAA[c]		98
T3	ATTTTGAAGAGAAATACTTTAAATCAAATGATGAAGATATAGCGTCTAATCTAT GAGGGTGGGTAGGGTGGGTGTAGTACAGGTCGTGCCGCCAAAAA		98
T4	TGTAGTACAGGTCGTGCCGCCAAAAA		26
P1	ppp*GGCGGCACG*ACC[d]		12
T5	GTCTGGAATGATGAAGATTACTAGTGAAGATTCTGAGCGTCTTAATCTAAGCA CTCGCTATGTTTTCAAGTTT		73
T6	AATGATGAAGATATCTGGTCGCTCCATTCTGGAGCGACCTCTTAATCTAAGCAC TCGCTATGTTTTCAAGTTT		73
T7	GTCTGGAATGATGATGAGGGTGGGTAGGGTGGGTGAGCGTCTTAATCTAAGCA CTCGCTATGTTTTCAAGTTT		73
P2	/Cy5/CTTGAAAACATAGCGA		16
T8	/Biotin/AATCTAGTAACATAGTATACATAAGCGCTCCAGGC	Binding kinetics	35
P3	*GCCUGGAG*CG/3ddC/[e]		11
T9	AATCTAGTAACATAGTATACATAGGCGCTCCAGGC	Cryo-EM	35
P4	*GCCUGGAGC*G/3ddC/		11

[a] Sequences are listed in order from 5'-end to 3'-end.
[b] The regions complementary to a primer are underlined.
[c] The structured regions of a DNA template are in red (T2 and T6 form a hairpin, T3 and T7 form a G-quadruplex).
[d] The ribonucleotides are in italics; ppp indicates the 5'-triphosphate group.
[e] 3'-dideoxy-cytidine.

Supplemental Table S2. Cryo-EM data collection, refinement and validation statistics for the RPAcore/Polα$_{CD}$/DNA complex.

**Data collection and processing**	
Microscope	Glacios
Voltage (keV)	200
Detector	Falcon 4i
Magnification	165,000
Electron dose (e^{-}/Å^{2})	60
Pixel size (Å)	0.72
Defocus range (µm)	-0.8 to -2.4
Symmetry imposed	C1
Resolution (Å)	3.5
FSC threshold	0.143
**Refinement**	
Initial model used (PDB code)	4QCL, 1L1O
Non-hydrogen atoms	11919
Protein residues	1378
RNA/DNA nucleotides	42
Cofactors/ions	3
R.m.s. deviations	
Bond lengths (Å)	0.003
Bond angles (°)	0.61
**Validation**	
MolProbity score	1.6
Clash score	5.9
Poor rotamers (%)	0.65
Ramachandran plot (%)	
Favored	96.11
Allowed	3.81
Disallowed	0.07
Fit to map (CC_{mask})	0.83
**Accession codes**	9MJ5, EMD-48312

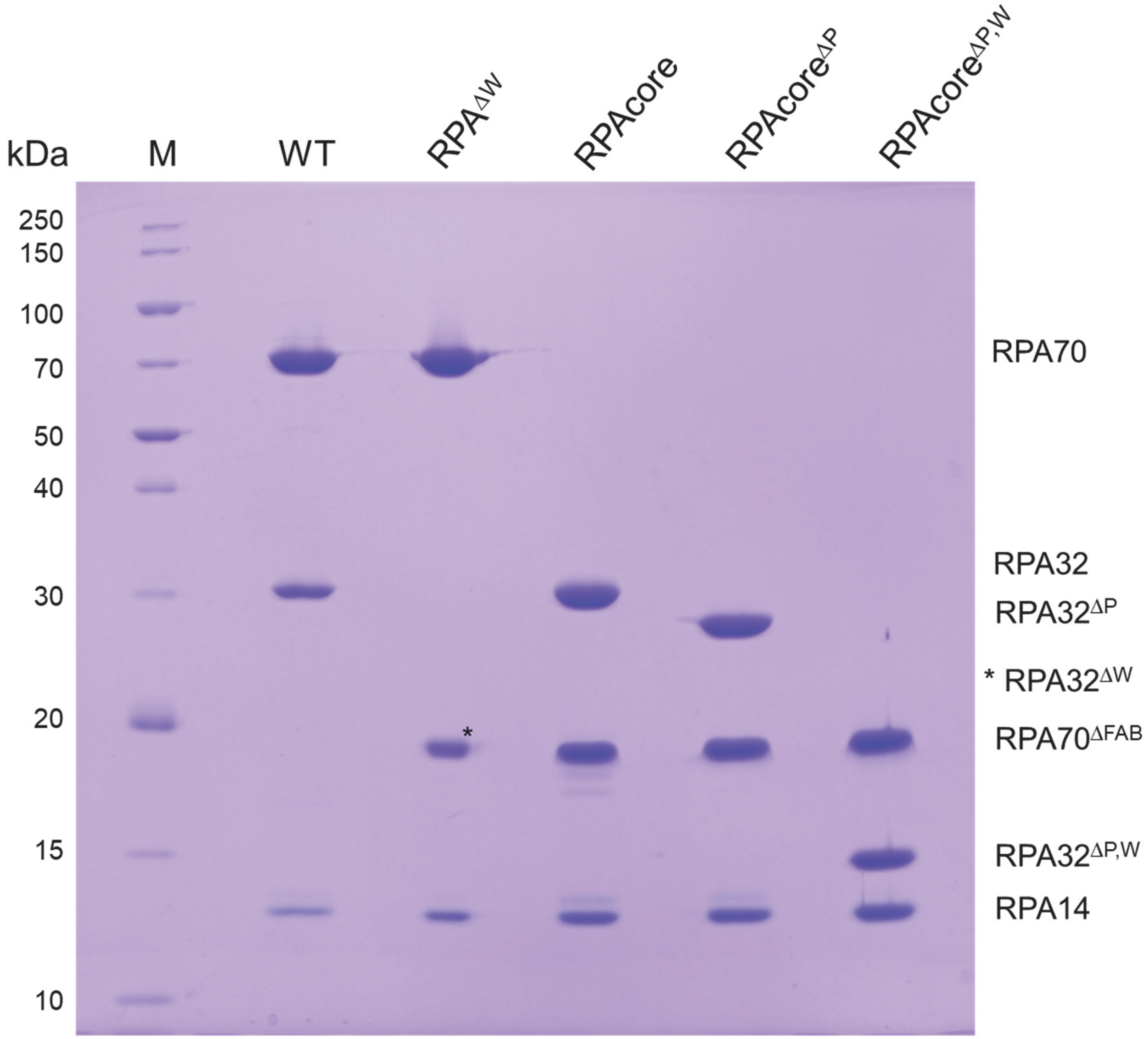

Supplemental Figure S1. Analysis of purity of human RPA and its variants. Proteins were separated by 13% SDS-PAGE and stained by Coomassie Brilliant Blue R-250.

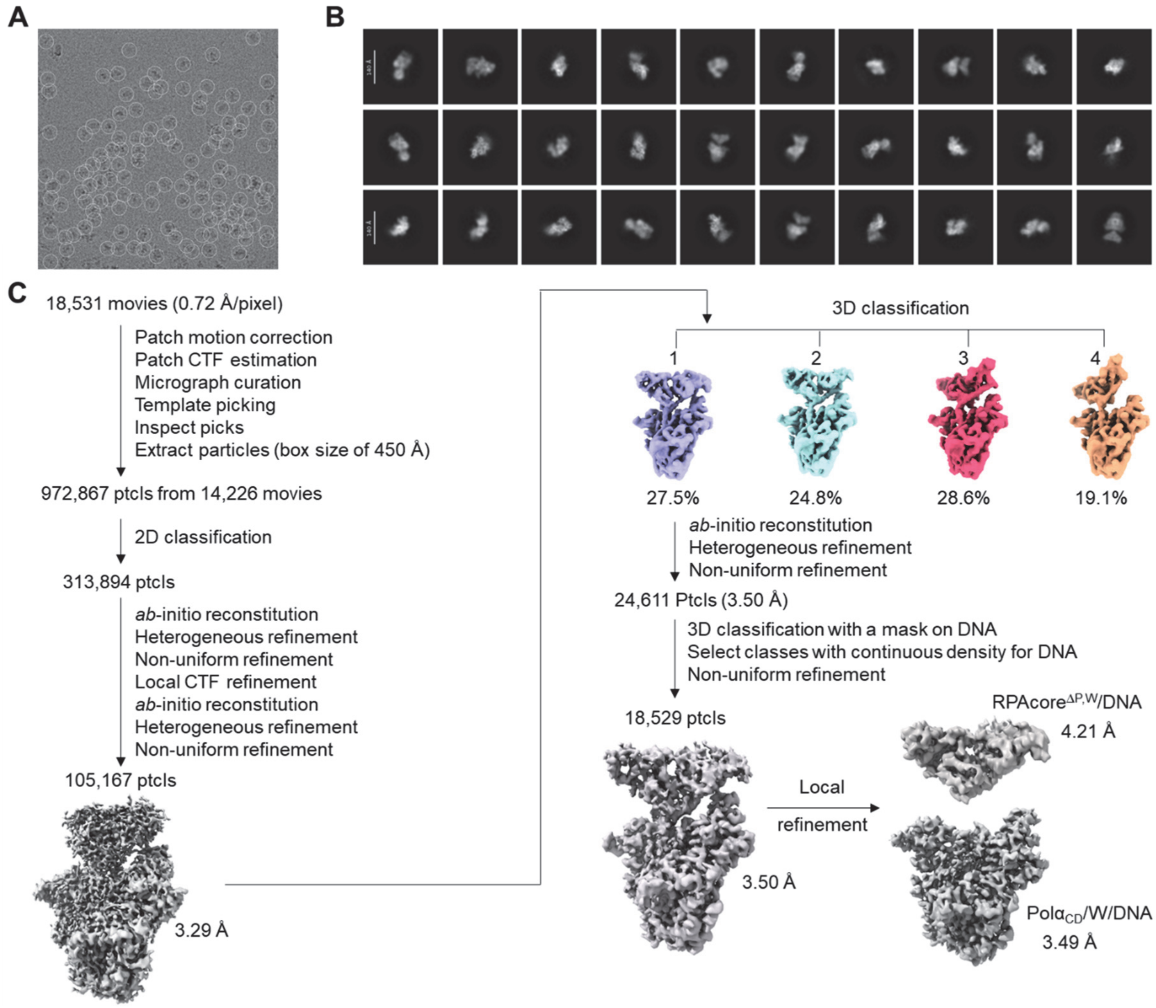

Supplemental Figure S2. Cryo-EM processing pipeline for the RPAcore/Polα_{CD}/DNA complex. (A) Representative micrograph with particles (ptcls) passed inspection before extraction. (**B)** 2D classification averages selected after the third round. (**C)** Cryo-EM processing pipeline.

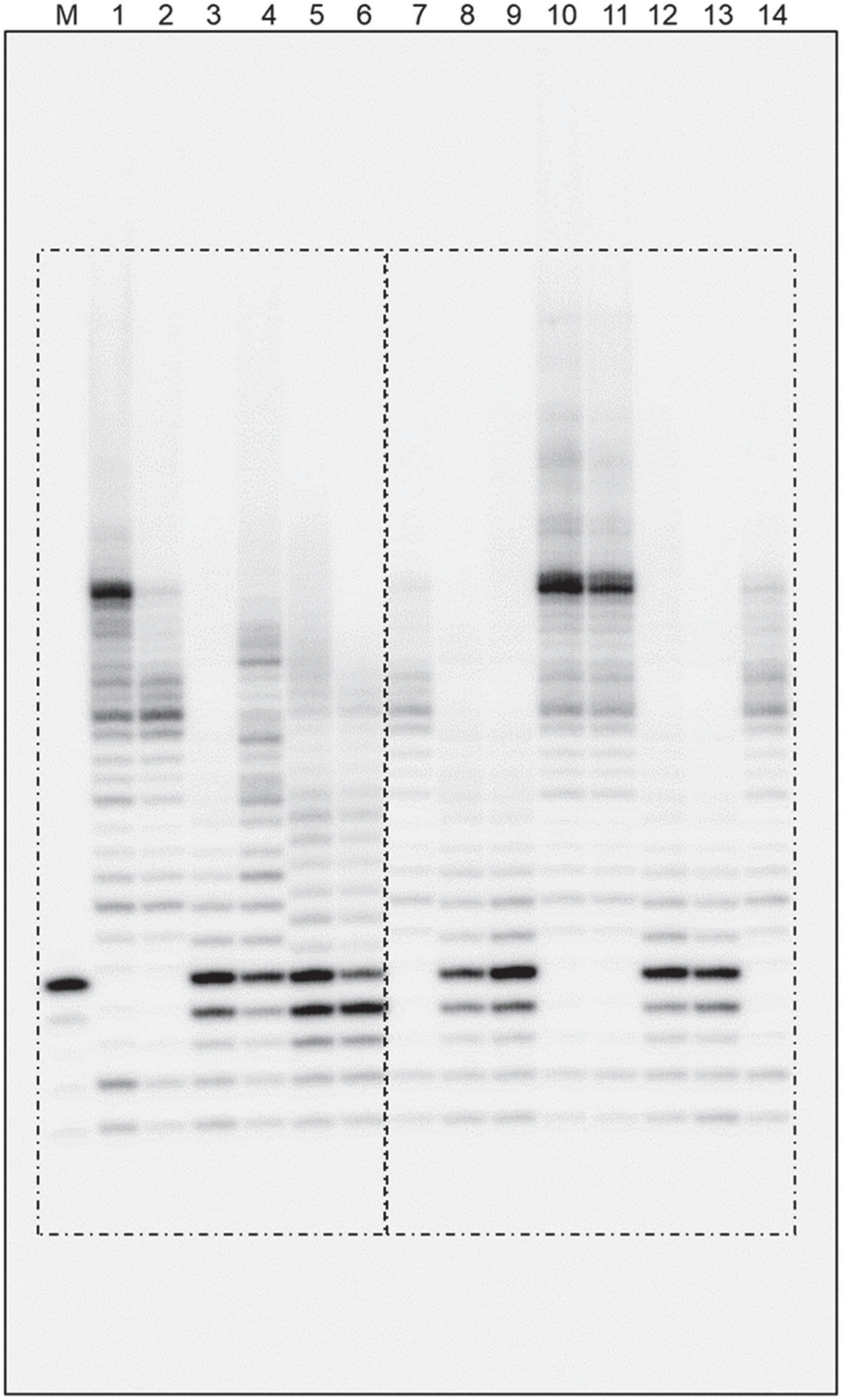

Supplemental Figure S3. The original gel image used for making panels A and B of Figure 2.

Selected areas correspond to panels A and B.

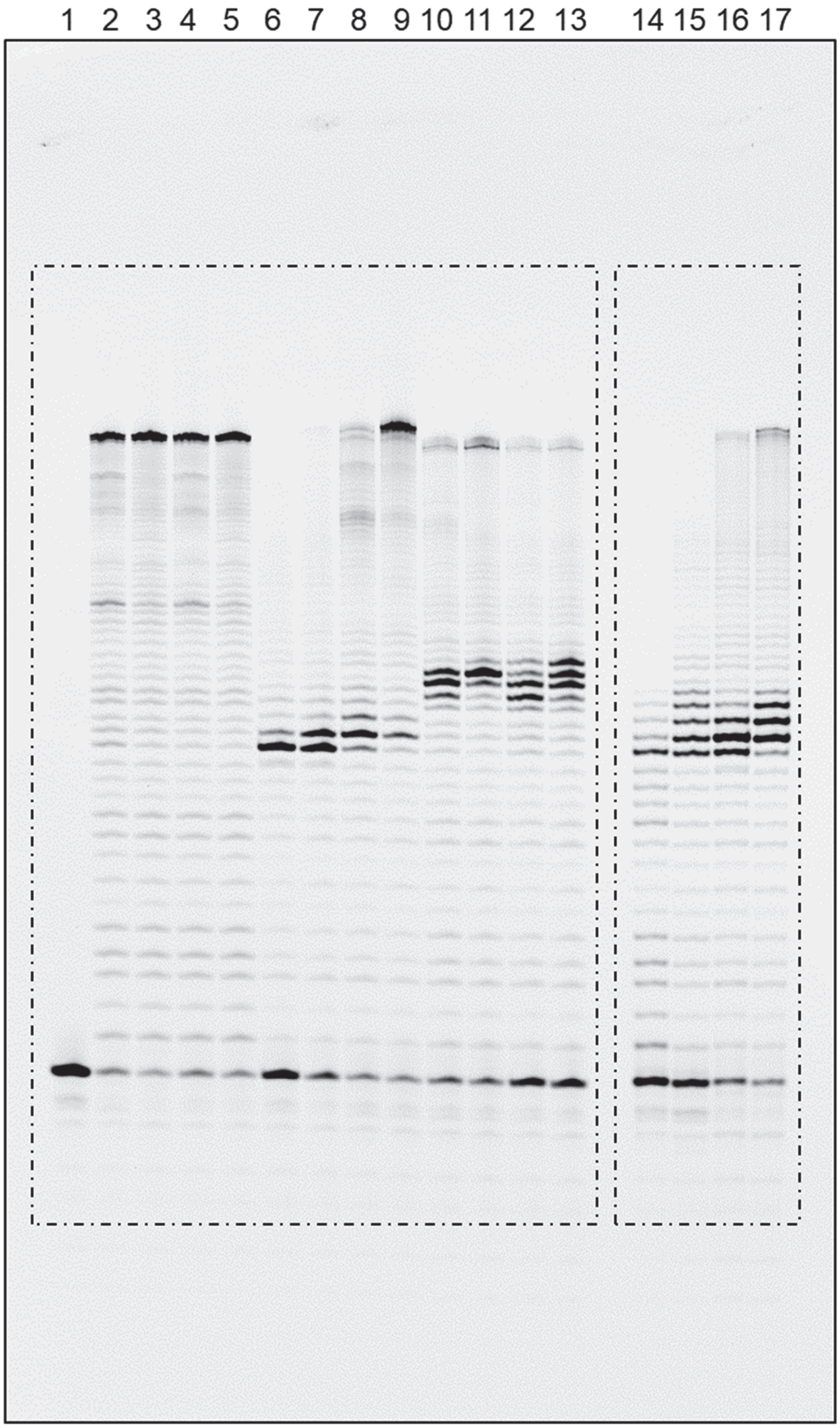

Supplemental Figure S4. The original gel image used for making panels A and B of Figure 3. Selected areas correspond to panels A and B.

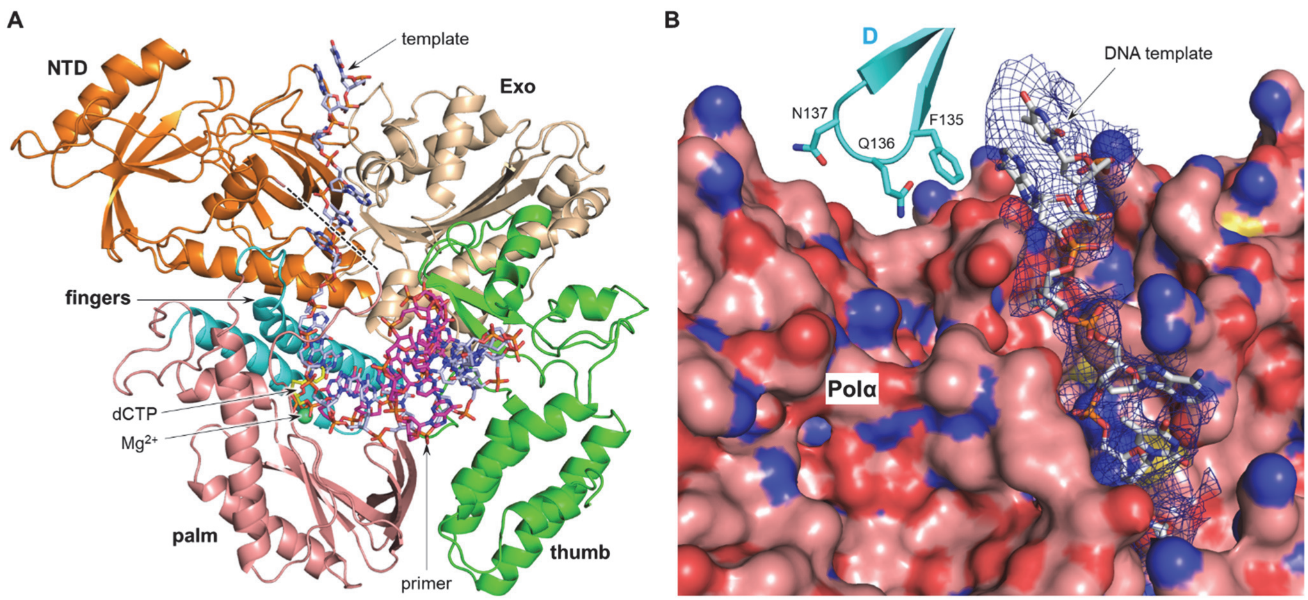

Supplemental Figure S5. The cleft between the N-terminal and exonuclease domains of Polα directs a DNA template from RPA to the Polα active site. **(A)** The overall view of Polα/DNA/dCTP complex. Polα domains N-terminal (NTD), exonuclease (Exo), fingers, palm, and thumb are represented as cartoon and colored orange, wheat, cyan, salmon, and green, respectively. Template, primer, and dCTP are shown as sticks with carbons colored light-blue, magenta, and yellow, respectively. The disordered linker (residues 810-831) connecting the N-terminal and palm domains is represented by dashed line. RPA and DNA in complex with it are not shown for clarity. (**B**) The close-up view of a DNA template in the Polα cleft and the Polα-RPA contact area formed by NTD and the D-domain. Polα and DNA are represented as surface and sticks, respectively. Three RPA residues in proximity to Polα NTD are shown as sticks. Atoms of nitrogen, oxygen, sulfur, and phosphor are colored blue, red, yellow, and orange, respectively. Carbons of protein and DNA are colored salmon and light-blue, respectively. Electronic density of DNA is shown as a blue mesh.

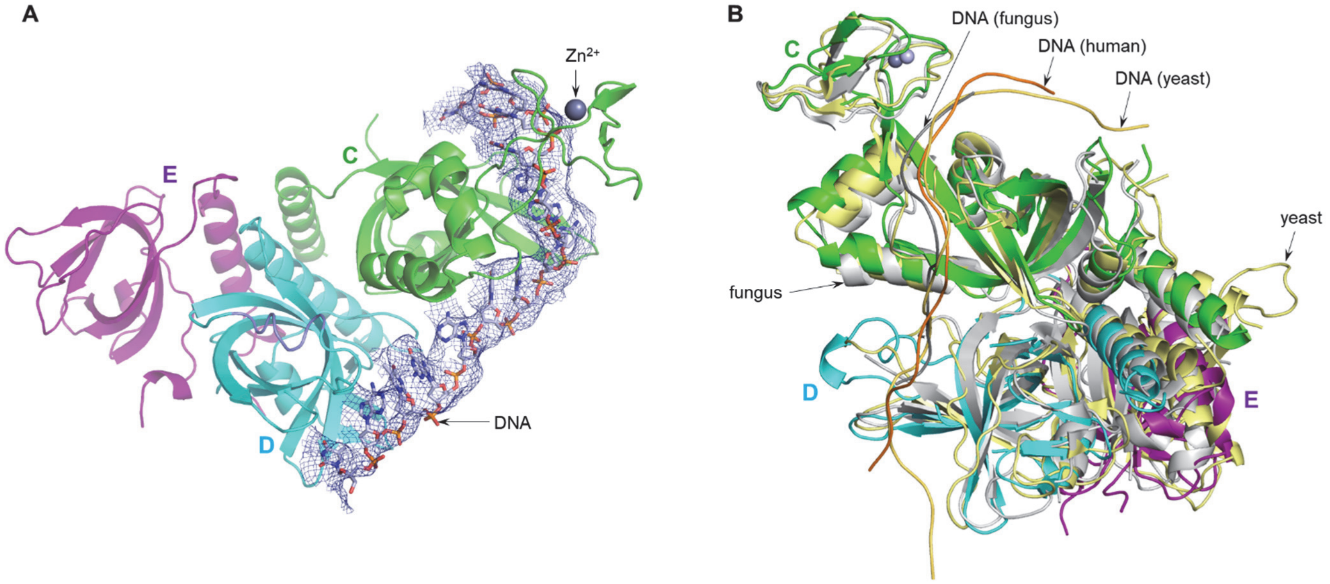

Supplemental Figure S6. Human RPAcore binds DNA similar to other species. (A) DNA interacts with the C- and D- domains of human RPAcore. DNA and its electronic density are shown as sticks and blue mesh, respectively. Protein is represented as cartoon at 20% transparency. **(B)** Comparison of a DNA path on RPAcore from different species. The human RPAcore/DNA complex was aligned with corresponding complexes, containing yeast (pdb ID 6I52) and fungal RPA (pdb ID 4GNX), with rmsd of 2.3 Å and 2.6 Å, respectively. DNA is represented as ribbon. The C, D, and E domains of human RPAcore are colored green, cyan, and purple, respectively; DNA is colored orange. The yeast and fungal RPAcore molecules (and DNA in the complex with them) are colored pale-yellow and gray, respectively. Zn^{2+} ions in human and fungal RPA are shown as spheres and colored light-blue and gray, respectively. For clarity, the AB-domains of fungal RPA as well as DNA in complex with them are not shown.

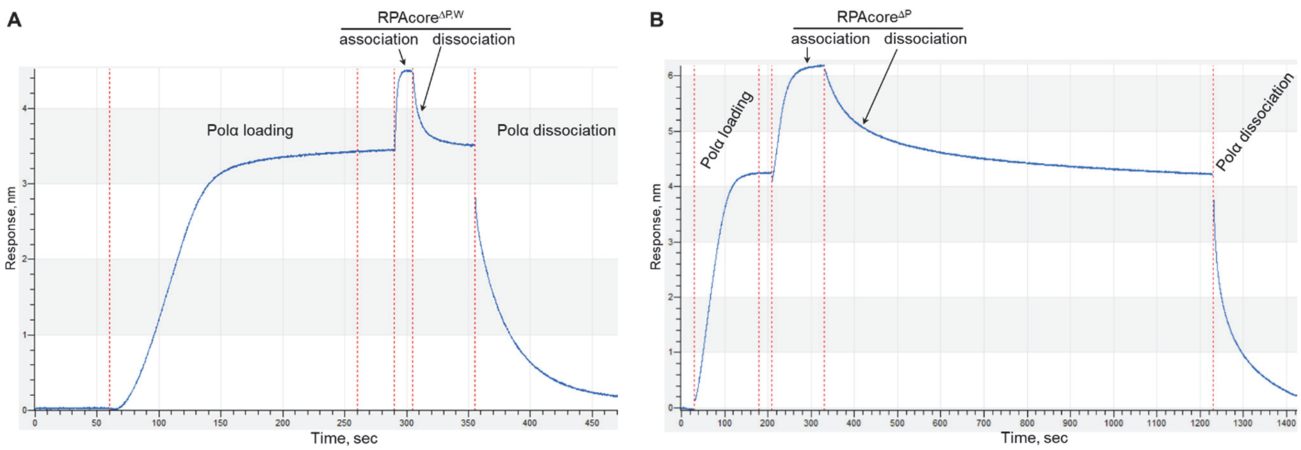

Supplemental Figure S7. DNA-binding studies of RPAcore$^{\Delta P,W}$ (A) and RPAcore$^{\Delta P}$ (B) in the presence of Polα$_{CD}$. RPAcore$^{\Delta P}$ and its variant missing the W-domain dissociate from the DNA/Polα sensor in 15 min and 50 sec, respectively.

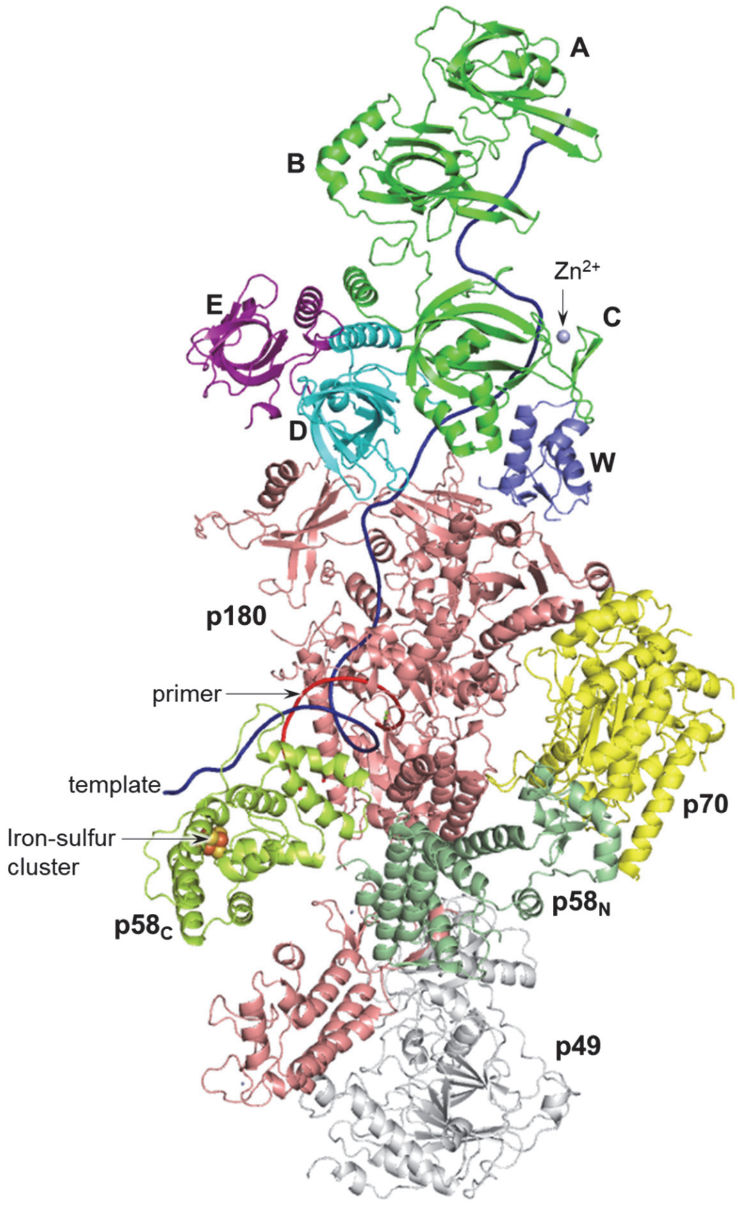

Supplemental Figure S8. The model of RPA/primosome elongation complex. The complex RPAcore/Polα$_{CD}$/DNA was superimposed onto the primosome elongation complex (pdb ID 8D9D) using Polα$_{CD}$ for alignment (rmsd of 0.56 Å). The model for human RPA in complex with DNA was generated using AlphaFold 3 Server and superimposed onto the modeled complex RPAcore/primosome/DNA using RPAcore for alignment (rmsd of 0.94 Å). From the RPA/DNA model generated by AlphaFold, only the AB-domains and 11-mer DNA in complex with them are shown. The F-domain of RPA is very flexible and is not shown for clarity. Human Polα comprises subunits p180 (catalytic) and p70 (accessory); human primase comprises subunits p49 (catalytic) and p58 (accessory), which has N-terminal (p58$_{N}$) and C-terminal (p58$_{C}$) domains. p58$_{C}$ interacts with the primer 5'-end and regulates all steps of RNA-DNA primer synthesis.

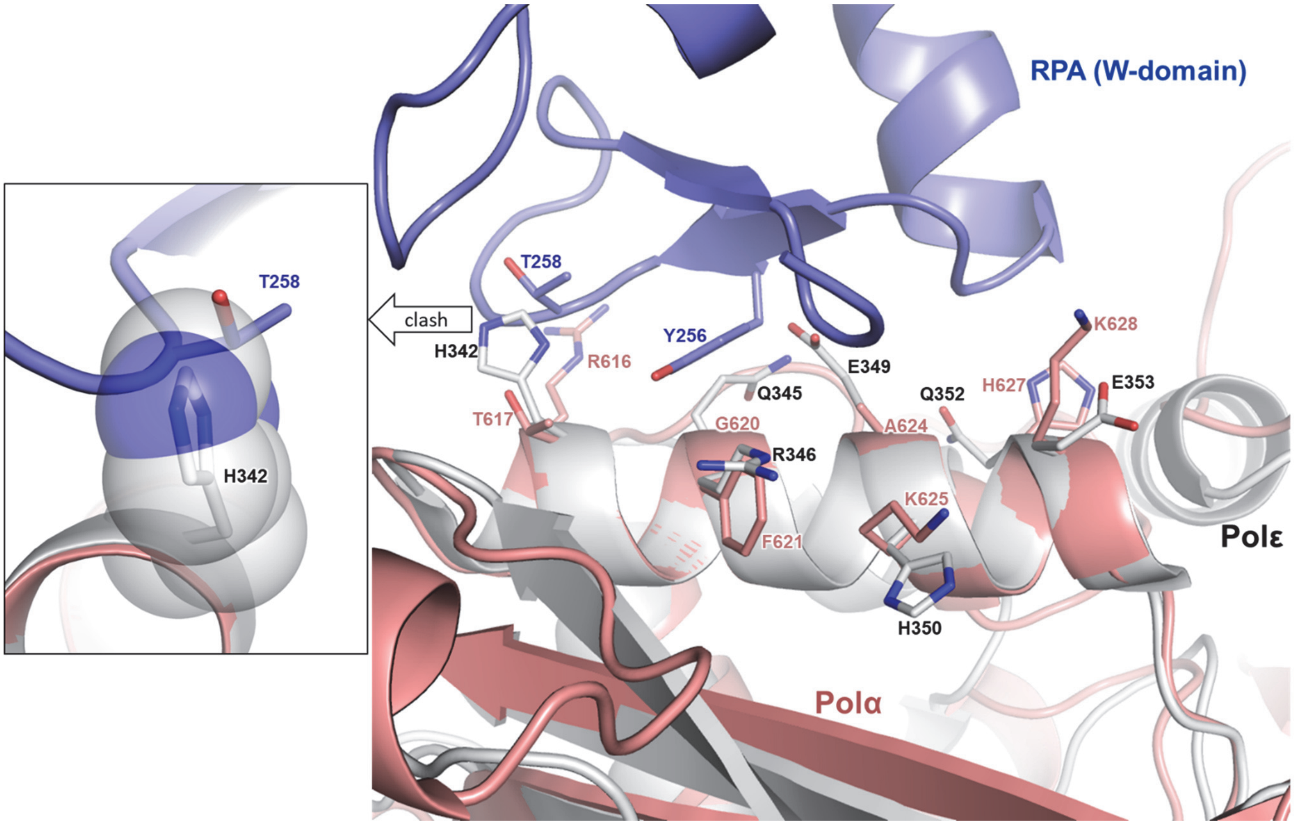

Supplemental Figure S9. Human Polε cannot interact with the W-domain of RPA in the same way as Polα. The complex RPAcore/Polα$_{CD}$/DNA was superimposed onto the Polε/PCNA/DNA complex (pdb ID 9F6E) using the Polα residues 612-655 for alignment. A close-up view of the potential clash between His342 of Polε and Thr258 of RPA32 is shown on the left (the atoms of His342 are shown as spheres with a 50% transparency). The residues are represented as sticks, with the carbons of RPA, Polα, and Polε colored slate, salmon, and gray, respectively.

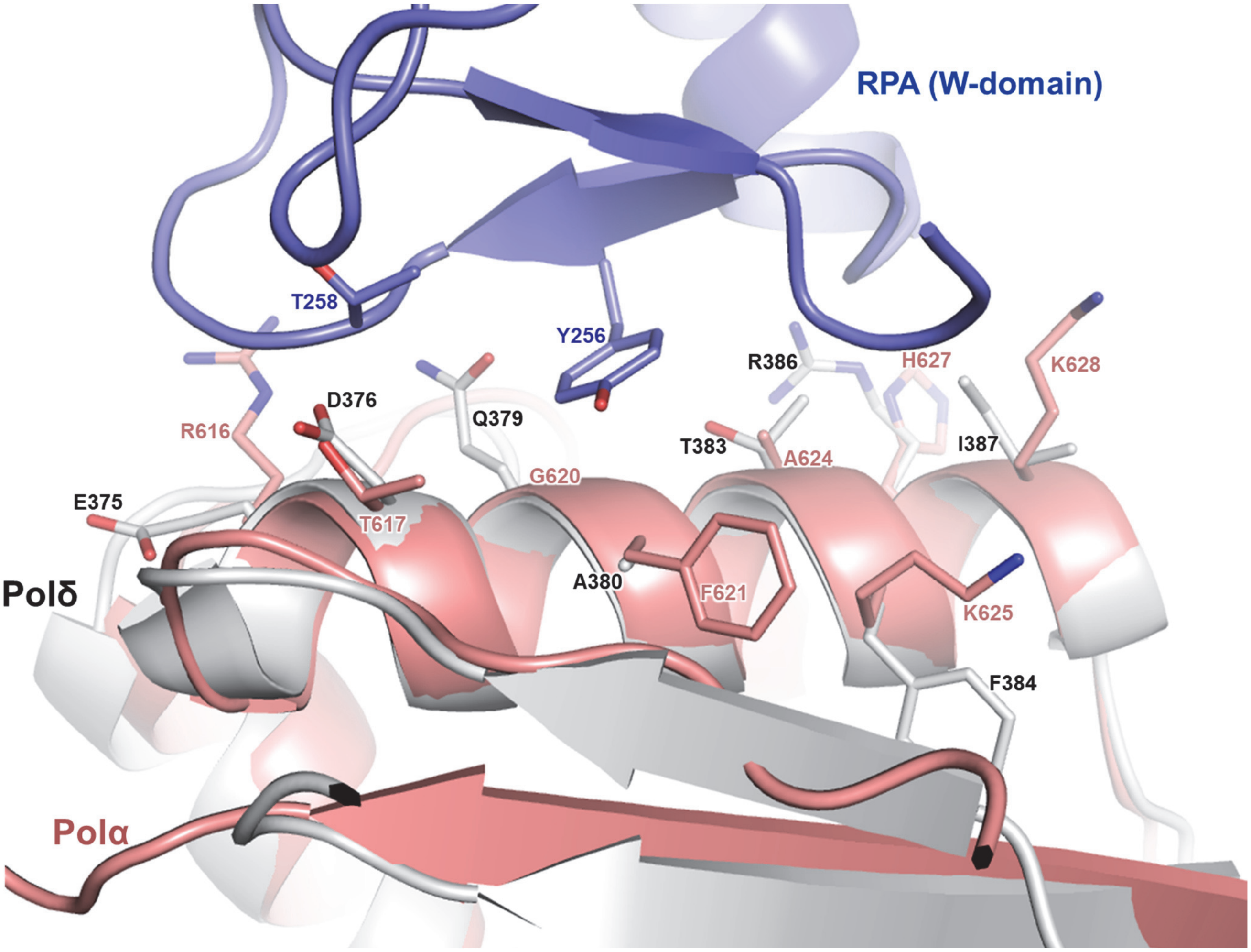

Supplemental Figure S10. There are no steric impediments for the interaction between human Polδ and the W-domain of RPA. The complex RPAcore/Polα$_{CD}$/DNA was superimposed onto the Polδ/PCNA/DNA complex (pdb ID 6TNY) using the Polα residues 612-655 for alignment. The residues are shown as sticks, with the carbons of RPA, Polα, and Polδ colored slate, salmon, and gray, respectively. Polδ residues Gln379 and Arg386 have the potential for H-bond formation with RPA. Polδ residues Gln379, Ala380, Thr383, and Ile387 are poised for hydrophobic interactions with RPA.

Supplemental Movie S1. Movement of RPAcore$^{\Delta W}$/DNA relative to Polα$_{CD}$/W/DNA in an RPAcore/Polα$_{CD}$/DNA complex. The movie was generated using the morphing option of PyMol software and the coordinates of RPAcore/Polα$_{CD}$/DNA conformers with the largest difference in positions of RPAcore/DNA (conformers 1 and 4 in Supplemental Figure S2). The protein domains and DNA were color-coded as in Figure 4.
