# Supplementary material for: Explicit Scale Simulation for analysis of RNA-sequencing count data with ALDEx2
Source: NAR Genom Bioinform. 2025 Aug 19;7(3):lqaf108. doi: 10.1093/nargab/lqaf108 (PMC12362245; doi:10.1093/nargab/lqaf108)
Supplement: lqaf108_Supplemental_Files [file lqaf108_supplemental_files.zip › supplement_figs.pdf]

Supplement: Explicit Scale Simulation for analysis of RNA-sequencing count data with ALDEx2

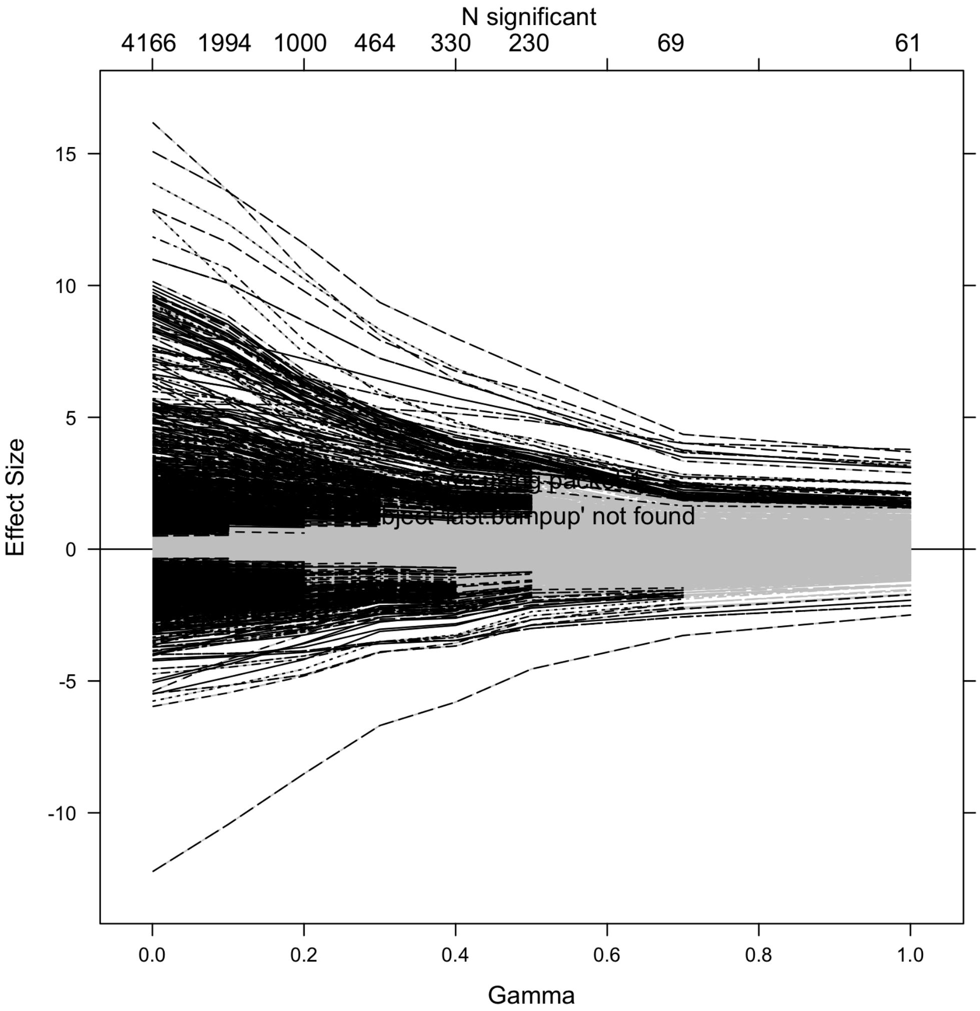

Supplementary Figure 1: The aldex.senAnalysis() function was used with the yeast transcriptome dataset to generate outputs with γ values of 1e-3, 0.1, 0.2, 0.3, 0.4,0.5, 0.7 and 1. The plotGamma() function was used to plot the result. Transcripts that are statistically significant are shown in black, and if not significant are in grey. The γ values, standardized effect size and number of significant transcripts at each value are given on the axes.

Supplementary Figure 1 shows that root cause of the large number of significant parts in this dataset is the very low dispersion of transcripts. Here we see a graphical output from the *aldex.scaleSim()* function with the yeast transcriptome dataset described in the text (1, 2). This allows us to examine which transcripts are sensitive to even minimal amounts of scale uncertainty using $\gamma = (1e-3, 0.1, 0.2, 0.3, 0.4, 0.5, 0.7, 1)$. Adding scale uncertainty increases the minimum dispersion in the analysis. Here it is obvious that even a negligible amount of scale uncertainty $\gamma = 0.1$ removes over half of the transcripts that were formerly significantly

different, and all of these had very small effect sizes.

We recommend a minimum scale uncertainty of between 0.2 and 0.5, and suggest that the *aldex.senAnalysis()* function be run on all datasets to identify transcripts that are sensitive to small γ values. The individual *aldex()* outputs can be accessed as sequential entries in the list output, or the analysis as a whole can be plotted with the *plotGamma* function.

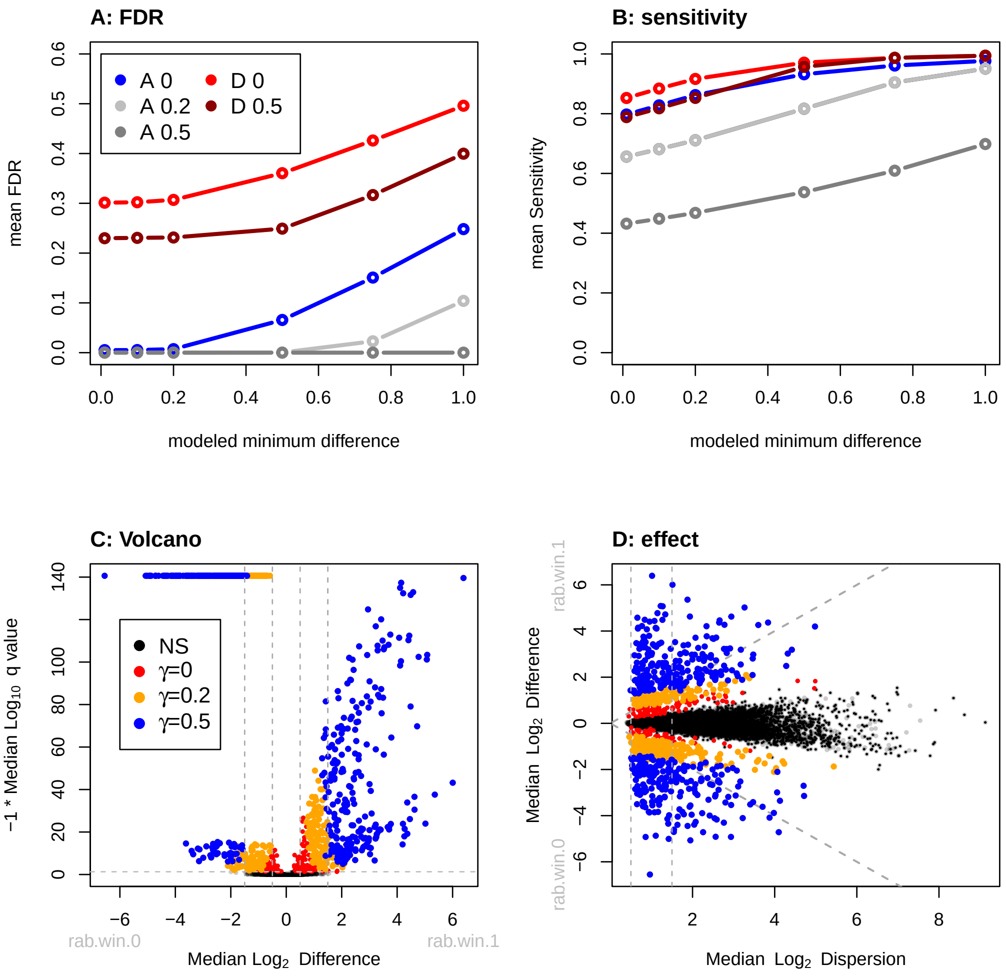

Supplementary Figure 2: Adding scale to the BRCA dataset reduces false positives in the same way as the PD-1 dataset.

Supplementary Figure 2 shows an analysis of synthetic data to identify the FDR and sensitivity with a standard of truth in modeled data. This second dataset is the BRCA1 dataset from Li et al. (3) which is a transcriptome analysis of breast cancer and matched tissue controls from The Cancer Genome Atlas collection (4) with the count tables collected from the Li et al. supplementary dataset. Transcripts that were differentially abundant at different gamma values are plotted. The first panel in the top row shows FDR at different γ (for ALDEx2) and fold-change cutoffs (for DESeq2) and the second panel in the top row does the same for sensitivity. It is noteworthy that in this dataset the FDR for DESeq2 is still very high and that ALDEx2 behaves similarly to the PD-1 dataset. The sensitivity for both tools is much higher and adding a small amount of scale uncertainty strongly controls the FDR while having minimal impact on sensitivity. The panels in the bottom row show volcano and effect plots of the outputs for ALDEx2 and recapitulate the results of Figure 3 in the text.

The first panel in Supplementary Figure 3 shows that the lowest dispersion transcripts are differentially affected by adding scale uncertainty. The graph in this first panel supports the concept that there is not a

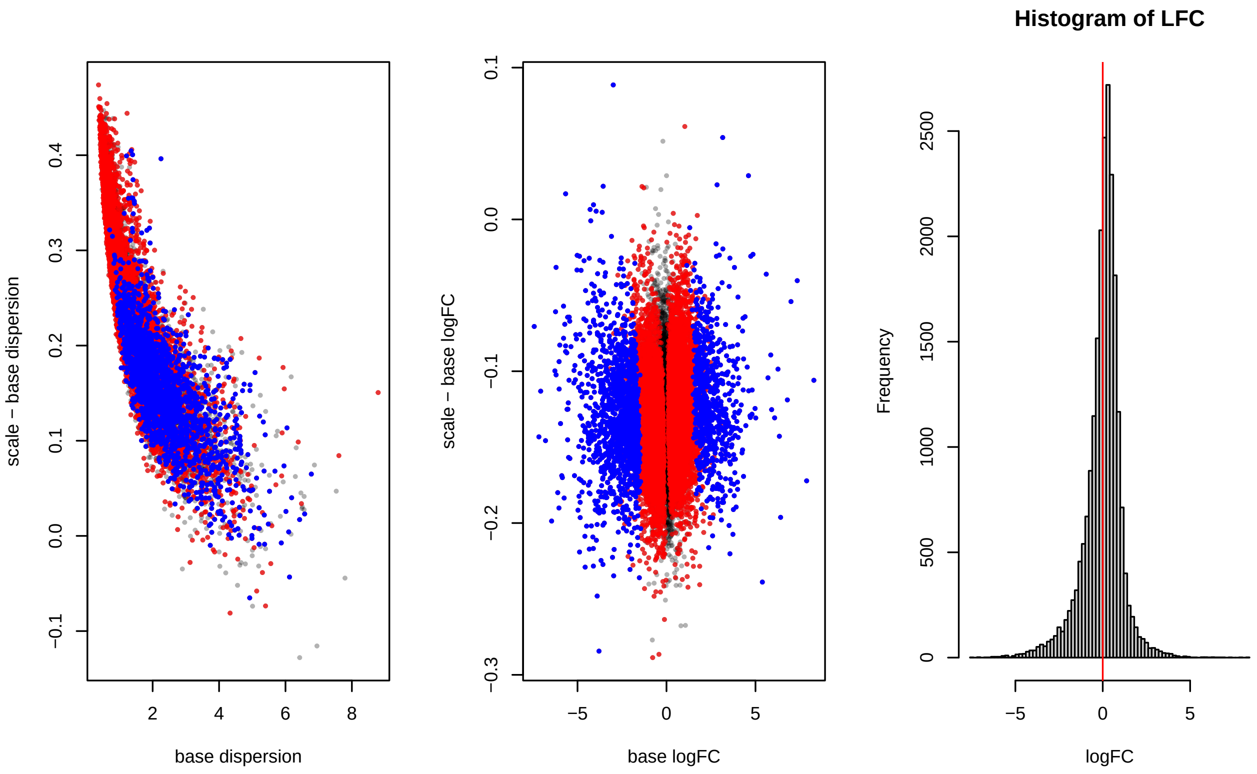

Supplementary Figure 3: Adding scale reduces positive identifications in biological replicate experiments; data from the Li et al. 2022. The sensitivity analysis in the plot on the left shows that many transcripts are excluded, even when the gamma value is very low. The colors are the same as in Supplementary Figure 1. The effect plot on the left shows that those transcripts with low difference between and low dispersion are not identified when scale is included (blue) vs. when scale is not included (blue or red). The middle plot shows that those transcripts where the difference in dispersion between the scaled and unscaled analysis is largest are most affected by adding scale. This plot and the next, color transcripts identified as signficant (FDR less than 0.05) when scale is included (blue) vs. when scale is not included (blue and red). The right-hand histogram shows that the centre of the data is not on 0 fold-change between groups, and so a full scale model would likely be useful.

defined fold-change cutoff when $\gamma = 0.5$, but rather it is a dynamic between fold-change and dispersion and is consistent with the result seen in Figure 3 in the main text in a different dataset. The second panel shows that the log-fold change is only affected minimally by adding scale. The final panel shows that there is a slight scale mis-specification, in that the median difference between groups is not centered exactly at 0, and so this dataset may benefit from a full (informed) scale model. This analysis was again conducted using the BRCA1 dataset.

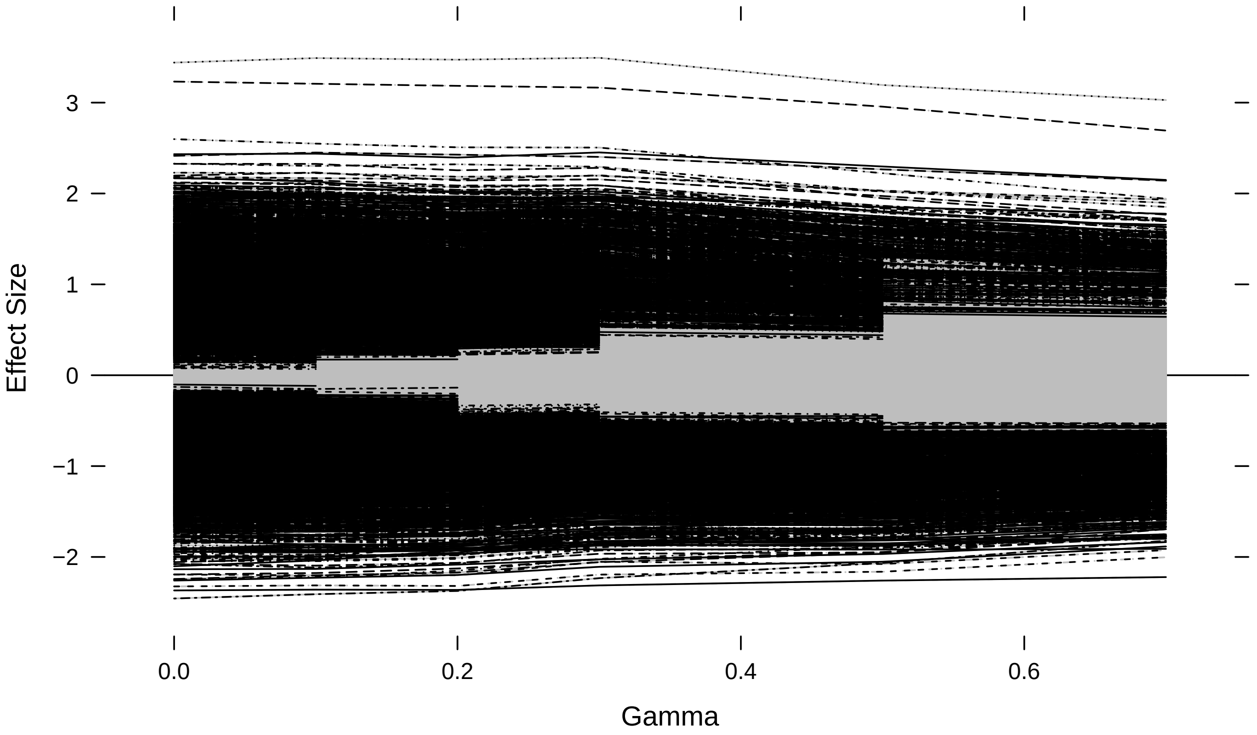

Supplementary Figure 4: Adding scale uncertainty reduces positive identifications in biological replicate experiments; data from the BRCA dataset obtained from Li et al. 2022. The sensitivity analysis plot shows that many transcripts are excluded, even when the gamma value is very low. This is an indication that these transcripts are extremely sensitive to normalization assumptions and are likely to be FP. The colors are the same as in Supplementary Figure 1.

Supplementary Figure 4 shows a sensitivity analysis of the BRCA1 dataset. In the when $\gamma = 1e^{-3}$ there are 15841 significant transcripts (72% of all 21813 transcripts!), which drops to 12167 when $\gamma = 0.1$ and 7737 when $\gamma = 0.2$, 5055 when $\gamma = 0.3$, 2096 when $\gamma = 0.5$ and finally 1204 when $\gamma = 0.7$.. Here there is a sharp drop in significant transcripts betwee $\gamma = 0.1, 0.3$ and then relative stability between 0.3 and 0.5, suggesting that these latter γ values are likely to be appropriate. Note that in biologically-replicated data adding a small amount of scale uncertainty has less of an effect at small uncertainty values than in technically replicated data.

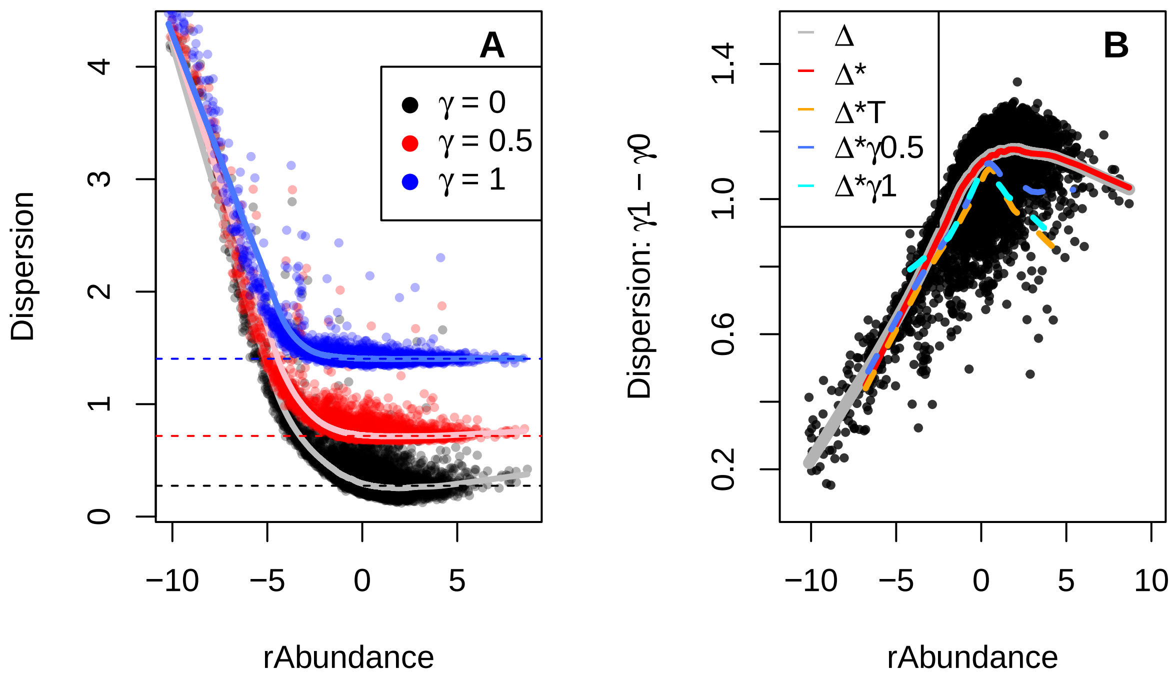

Supplementary Figure 5: Adding scale uncertainty changes the dispersion distribution. Panel A shows a plot of the expected value for relative abundance vs the expected value for the pooled dispersion as output by `aldex.effect`. The dashed horizontal lines show the median value for the features with a rAbundance between -0.5 and 0.5, and the light colored lines are lowess lines of fit through the center of mass of the data. Panel B plots the dispersion difference between $\gamma = 1$ and $\gamma = 0$; note the non-linear relationship that highlights the rotation that is evident in Panel A. The colored lines indicate the lowess line of fit through the centre of mass of the plot for the various populations of points. The thick grey line is the total population and shows the difference Δ, the red line is the population of significant transcripts (*) with $\gamma = 0$, the dashed orange line is the population of significant transcripts with a difference threshold (T) of about $\pm 2^{1.4}$-fold change, the dashed blue line is the population of significant transcripts with $\gamma = 0.5$, and the dashed cyan line is the significant population with $\gamma = 1$. Δ: Difference, *: significant, T: thresholded.

The effect on dispersion with increasing amounts of scale uncertainty in the technical replication dataset (yeast) are shown in Supplementary Figure 5A, where we can see that the dispersion increases as uncertainty is added. Note that the dispersion in the unscaled analysis in Figure 5A reaches a minimum near the mid-point of the distribution, and also does so when the analysis is conducted with DESeq2 (Supplementary Figure 5). This shows more clearly that dispersion of many transcripts is almost negligible in the absence of scale uncertainty. This plot makes the counter-intuitive suggestion that the variance in expression of the majority of genes with moderate expression is more predictable than highly-expressed genes or of housekeeping genes (5). This is at odds with the known biology of cells where single cell counting of highly-expressed transcripts shows that they have little intrinsic variation (6, 7).

Adding scale uncertainty by setting $\gamma = 0.5$, or $\gamma = 1.0$, increases the minimum dispersion as shown in Figure 5A by the red and blue data points, and by the colored lines of fit through the centre of mass of the data. Less obvious is that the additional dispersion is not applied equally to all points. Figure 5B shows a plot of the difference between the $\gamma = 0$ and $\gamma = 1$ data and here we can see that scale uncertainty is preferentially increasing the dispersion of the mid-expressed transcripts that formerly had negligible dispersion; examine the grey line of best fit (overlaid by the red line) for the trend. Panel B also shows the trend of the expression-dispersion relationship for transcripts that are classed as statistically significant. The red line shows the trendline with no added scale uncertainty, and this trendline exactly overlays with the grey trendline for the bulk of transcripts. The orange trendline indicates those transcripts that are both statistically significant and that have a thresholded expression level of ± 1.4, and the dark blue and cyan lines show the statistically significant trendline for $\gamma = 0.5$, or 1.0.

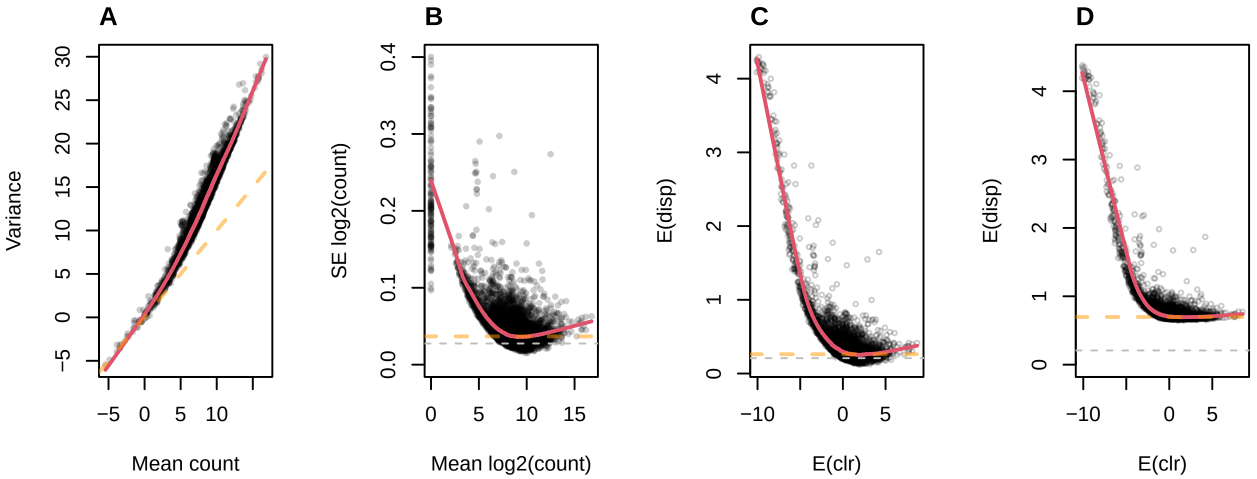

Supplementary Figure 6: Plot of abundance v dispersion for the yeast transcriptome dataset as counts, as logarithms of counts, and as CLR values. Panel A shows that the data are over-dispersed relative to a Poisson distribution which is represented by the dashed line when plotted on a log-log scale. Panel B shows that the relationship between the mean and the dispersion calculated in DESeq2, here the standard error (SE) of the mean, is very different when the data are log-transformed first. Panel C shows the equivalent values calculated by ALDEx2 in which the expected CLR value for each transcipt are plotted vs. the expected dispersion. Panel D shows the output for ALDEx2 with $\gamma = 0.5$. The red line in each panel shows the LOESS line of fit to the mid-point of the distributions. In panels B and C the amount of dispersion reaches a minimum at moderate values. The dashed orange line in panel A is the line of equivalence, and in panel B and C is the minimum y value. The values below the dashed grey line in panels B and C represent those below the first decile of dispersion.

Supplementary Figure 6 shows that the informed models with 5% or 14% difference in location between groups and $\gamma = 0.5$ provide nearly the same output for q-values, effect sizes and difference between groups (black). The line of identity is given as the grey diagonal. However, using the default CLR values to specify location are very different. In the q-value and effect plots, there is multiple populations of points that indicate the Type 1 and 2 errors that occur when the location is not specified properly. In the difference between groups plot, we see only a shift in the location that corresponds to moving the mass of the data points down by about 2.2 units (See Figure 3 in the main text)

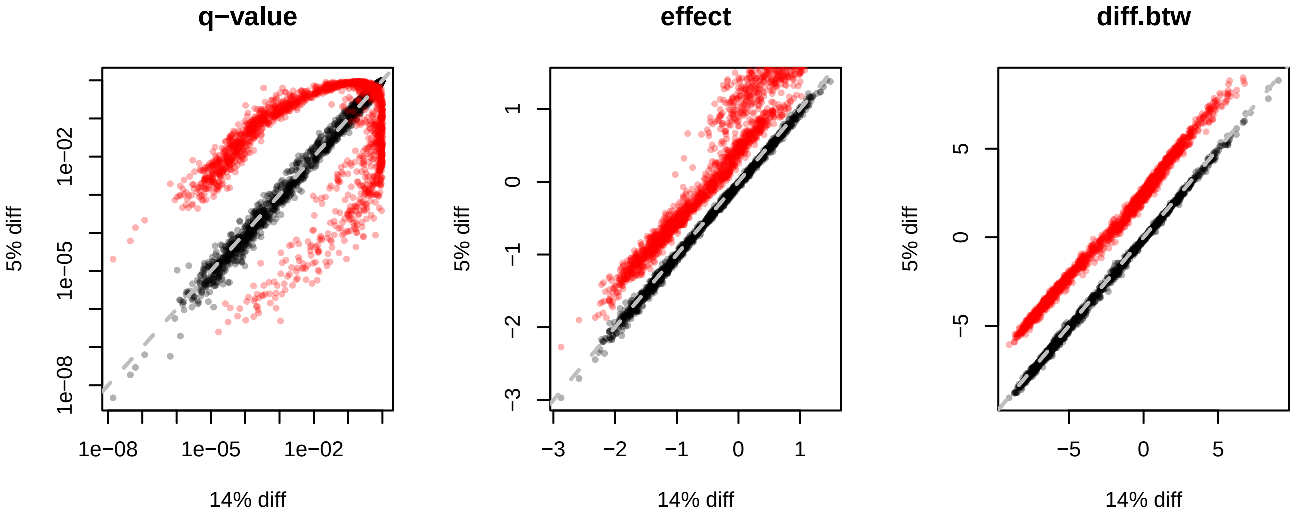

Supplementary Figure 7: Plots showing the similarity of outputs with different scale parameters. The black points show that using either a an informed model with 5% or 14% difference in location has a minimal effect on either the q-values, the effect size or difference between groups. In red, the same values are plotted with the default model that uses a naive estimate of the location derived from the CLR.

References

1. Gierliński,M., Cole,C., Schofield,P., Schurch,N.J., Sherstnev,A., Singh,V., Wrobel,N., Gharbi,K., Simpson,G., Owen-Hughes,T., *et al.* (2015) Statistical models for RNA-seq data derived from a two-condition 48-replicate experiment. *Bioinformatics*, **31**, 3625–3630.

2. Schurch,N.J., Schofield,P., Gierliński,M., Cole,C., Sherstnev,A., Singh,V., Wrobel,N., Gharbi,K., Simpson,G.G., Owen-Hughes,T., *et al.* (2016) How many biological replicates are needed in an RNA-seq experiment and which differential expression tool should you use? *RNA*, **22**, 839–51.

3. Li,Y., Ge,X., Peng,F., Li,W. and Li,J.J. (2022) Exaggerated false positives by popular differential expression methods when analyzing human population samples. *Genome Biol*, **23**, 79.

4. Cancer Genome Atlas Research Network, Weinstein,J.N., Collisson,E.A., Mills,G.B., Shaw,K.R.M., Ozenberger,B.A., Ellrott,K., Shmulevich,I., Sander,C. and Stuart,J.M. (2013) The cancer genome atlas pan-cancer analysis project. *Nat Genet*, **45**, 1113–20.

5. Rocha,D.J.P.G., Castro,T.L.P., Aguiar,E.R.G.R. and Pacheco,L.G.C. (2020) Gene expression analysis in bacteria by RT-qPCR. *Methods Mol Biol*, **2065**, 119–137.

6. Taniguchi,Y., Choi,P.J., Li,G.-W., Chen,H., Babu,M., Hearn,J., Emili,A. and Xie,X.S. (2010) Quantifying e. Coli proteome and transcriptome with single-molecule sensitivity in single cells. *Science*, **329**, 533–8.

7. Scott,M., Gunderson,C.W., Mateescu,E.M., Zhang,Z. and Hwa,T. (2010) Interdependence of cell growth and gene expression: Origins and consequences. *Science*, **330**, 1099–102.
